# Assessment of Knowledge of HIV/AIDS and Association With Socioeconomic Disparities Among Young Women in Low- and Middle-Income Countries, 2003 to 2018

**DOI:** 10.1001/jamanetworkopen.2020.35000

**Published:** 2021-01-22

**Authors:** Fan Yang, Zhihui Li, S. V. Subramianian, Chunling Lu

**Affiliations:** 1 Nanjing Medical University School of Health Policy and Management, Nanjing, Jiangsu Province, People’s Republic of China; 2 Department of Global Health and Population, Harvard T.H. Chan School of Public Health, Boston, Massachusetts; 3 Department of Social and Behavioral Sciences, Harvard T.H. Chan School of Public Health, Boston, Massachusetts; 4 Division of Global Health Equity, Brigham and Women’s Hospital, Boston, Massachusetts; 5 Department of Global Health and Social Medicine, Harvard Medical School, Boston, Massachusetts

## Abstract

**Importance:**

Knowledge of HIV/AIDS among the younger population is crucial for long-term control. Little research has been done on which groups of young women (aged 15-24 years) in low- and middle-income countries (LMICs) have been left behind with respect to obtaining this knowledge.

**Objective:**

To provide an up-to-date assessment of the levels of and changes in knowledge of HIV/AIDS and its associations with socioeconomic disparity among young women in LMICs.

**Design, Setting, and Participants:**

This cross-sectional study used nationally representative Demographic and Health Surveys with available data on HIV/AIDS knowledge in 51 LMICs between 2003 and 2018. The final data set contained 282 757 young women. For 40 LMICs with multiple surveys available, we examined changes in knowledge and socioeconomic disparities. Analyses of the survey data were conducted between December 1, 2019, and July 31, 2020.

**Main Outcomes and Measures:**

The percentage of young women who were able to (1) correctly identify the 2 major ways of preventing the sexual transmission of HIV, (2) reject the 2 most common local misconceptions about HIV transmission and (3) know that a healthy-looking person can transmit HIV. The level of and changes in young women’s knowledge of HIV/AIDS as well as knowledge disparities by residential area, wealth status, and level of education on the aggregate and country levels were assessed.

**Results:**

Of a total of 282 757 young women from 51 LMICs, 29.3% (95% CI, 24.1% to 34.5%) reported having knowledge of HIV/AIDS in the latest years, ranging from 1.0% (95% CI, 0.7% to 1.3%) in Afghanistan in 2015 to 64.9% (95% CI, 63.3% to 66.5%) in Rwanda in 2014 to 2015. Those living in rural areas, living in households in the lowest income quintile, or with low levels of education reported less knowledge about HIV/AIDS than their counterparts (−12.8 [95% CI, −10.6 to −14.9] percentage points; −21.8 [95% CI, −18.3 to −25.3] percentage points; and −19.4 [95% CI, −16.6 to −22.2] percentage points, respectively). Among the 40 countries that had undertaken at least 2 surveys during the period, the surveys indicated that there was a significant increase in knowledge of HIV/AIDS among young women in 24 countries (60.0%), but a significant decrease in 10 countries (25.0%). Less than half of countries experienced significant reductions in disparities associated with residential area (8 countries [20.0%]), wealth (5 countries [12.5%]), or education (17 countries [42.5%]).

**Conclusions and Relevance:**

This cross-sectional study of women in 51 LMICs found low levels and large gaps in knowledge of HIV/AIDS between groups, suggesting that future HIV-prevention campaigns should emphasize providing easily accessible information to socioeconomically disadvantaged groups.

## Key Points

**Question:**

How have knowledge of HIV/AIDS and associated socioeconomic disparities changed between 2003 and 2018 among young women aged 15 to 24 years living in low- and middle- income countries (LMICs)?

**Findings:**

In this cross-sectional study of 282 757 young women living in 51 LMICs, less than one-third reported having correct knowledge of HIV/AIDS; this disparity in knowledge was associated with rural residence, poverty, and low levels of education. Among young women living in the 40 LMICs with multiple surveys available during the study period, there was a significant increase in knowledge in 24 countries and a significant decrease in 10 countries.

**Meaning:**

These findings suggest that future HIV-prevention campaigns in LMICs should pay special attention to young women with disadvantaged socioeconomic status.

## Teaser

This cross-sectional study provides an up-to-date assessment of the levels of and changes in knowledge of HIV/AIDs and its association with socioeconomic disparity among young women in low- and middle-income countries.

## Introduction

Great progress has been made in reducing the prevalence of HIV.^1,2,3^ However, 37.9 million people worldwide were still living with HIV (including 1.7 million with new infections) in 2018, and 0.77 million AIDS-related deaths were reported in the same year.^1^ More than 90% of those with new infections were concentrated in low- and middle-income countries (LMICs).^1,4,5^ Controlling the HIV/AIDS epidemic requires the general population to have accurate information about prevention and transmission of the virus.^1,4^ As previous studies have demonstrated, knowledge of HIV/AIDS is an important determinant for success in promoting safe sex practices and changing behaviors that pose risks to health.^4,5,6,7^

Educating young people about HIV/AIDS has been an effective preventive measure, even in countries with limited health care provision.^5,6,8,9,10^ In 2016, the United Nations Political Declaration on Ending AIDS proposed that, by 2020, 90% of young people (aged 15 to 24 years) should have the knowledge to protect themselves from HIV. Indicators on HIV knowledge among young people are among the global indicators for measuring progress in the 2020 Fast-Track Commitments and Expanded Targets to End AIDS.^2,11,12,13^ The 2015 Millennium Development Goals report indicated that, at the regional level, fewer than 40% of young people aged 15 to 24 years old in sub-Saharan Africa had comprehensive correct knowledge of HIV/AIDS using the most recent data (up to 2014).^3^ Young women were particularly likely to have insufficient knowledge of HIV/AIDS and engage in risky sexual behavior: condom use among young women was 19 percentage points lower than among young men.^3^ Evidence about socioeconomic disparities among young women could help policy makers and other stakeholders develop effective interventions for closing the related knowledge gaps. Previous studies have only assessed changes in socioeconomic disparities in knowledge of HIV/AIDS among women of reproductive age (aged 15 to 49 years) in a few LMICS (eg, Bangladesh,^5^ Vietnam,^9^ and Cambodia^14^). Those studies found that disadvantaged populations were less likely to report knowledge of HIV/AIDS than their more advantaged counterparts. Little research has been done on country-level progress in reducing socioeconomic disparities among young women between 15 and 24 years. Using Demographic and Health Surveys (DHSs) from 51 LMICs, this study undertook a comprehensive, up-to-date assessment of the levels of and changes in reported knowledge of HIV/AIDS and associated socioeconomic disparities among young women between 2003 and 2018.

## Methods

### Data Sources

This study used nationally representative DHS data from 51 LMICs with available data on knowledge of HIV/AIDS between 2003 and 2018.^15,16,17^ DHS did not provide information relating to HIV/AIDS knowledge before January 1, 2003. Ethical approval for the DHS program was granted by the US Department of Health and Human Services. The DHS protocols and the process for obtaining oral informed consent for this study from all participants were approved by the ICF International institutional review board.^15^ No additional institutional review board approval was sought for this study, which followed the Strengthening the Reporting of Observational Studies in Epidemiology (STROBE) reporting guideline.

The DHSs are used to collect information on sociodemographic characteristics, child and maternal health, and awareness and behaviors associated with HIV/AIDS. Women aged 15 to 49 years were randomly selected through a multistage stratified design with equal-probabilistic sampling for each primary sampling unit (eAppendix in the Supplement).^16,17,18^ To ensure standardization and comparability across different sites and time, DHS adopted extensive interviewer training, an identical core questionnaire, and standardized measurement instruments and technologies. The response rate was greater than 90%.^15,16,17^

For consistency with the indicator proposed by the United Nations Political Declaration on Ending AIDS,^2,11,12,13^ we limited our analyses to young women aged 15 to 24 years. To obtain an up-to-date assessment on both the country and aggregate levels, we kept the countries with the most recent surveys conducted after 2010. We included 282 757 young women in the analysis of the most recent surveys in the 51 LMICs between 2010 and 2018 and 398 661 young women in the change analysis for the 40 LMICS who had undertaken at least 2 surveys between 2003 and 2018 (eFigure 1 and eTable 1 in the Supplement).

### Measures and Selection of Indicators

To measure a country’s level of knowledge of HIV/AIDS (indicator 5.1 for monitoring Fast-Track Commitment 5 and Expanded Targets to End AIDS by 2020^2,11,13^) among the population aged 15 to 24 years old, DHS constructed a survey module based on the definition of and knowledge about HIV prevention: identifying the 2 major ways of preventing the sexual transmission of HIV (using condoms and limiting sex to 1 “faithful, uninfected partner”), rejecting the 2 most common local misconceptions about HIV transmission, and knowing that a healthy-looking person can transmit HIV.^11,15^ In the DHS, if a respondent answered yes to the question, “Have you ever heard of an illness called AIDS?” she would then be asked to make selections (ie, yes, no, or do not know) for the following 5 statements:^15^ (1) “people can reduce the risk of contracting HIV by using condoms consistently,” (2) “people can reduce the risk of contracting HIV by only having sex with 1 faithful uninfected partner,” (3) “HIV cannot be transmitted by sharing food with people who have HIV,” (4) “a person cannot contract HIV from mosquito bites,” and (5) “healthy-looking people can have HIV.” To measure the percentage of young women who reported having knowledge of HIV/AIDS, those who provided positive answers to all 5 questions were included in the numerator, and all young women (15-24 years old) were included in the denominator.

### Disparity Measurements

The disparities in this study were measured by the absolute differences in the percentage of young women with knowledge of HIV/AIDS in the following 3 socioeconomic domains: residential area, wealth, and education. The DHS has a variable indicating a household’s wealth quintile based on its asset ownership. The methods for constructing wealth quintiles have been validated.^19,20^ Using a variable for level of education in the DHS, we divided the young women in each survey into 2 groups: those who received primary education or less and those who received more than primary education. We assessed inequality between the 2 subgroups in 3 socioeconomic dimensions: (1) the highest vs the lowest wealth quintiles, (2) those with higher education vs those with primary education or lower, and (3) those living in urban areas vs those living in rural areas. A greater than 0 difference indicated that individuals with lower socioeconomic status (SES) reported having a lower level of knowledge of HIV/AIDS, whereas a less than 0 difference indicated that the level of knowledge of HIV/AIDS was lower among populations with higher SES.^21^

### Statistical Analysis

We assessed the level of socioeconomic disparities for 51 countries with data for the most recent year since 2010 (ranging from 2010 to 2018). We also examined the changes in disparities over time for 40 countries with available data in multiple survey rounds. We performed the assessment on both the country and aggregate levels.

When generating country-level estimates of knowledge of HIV/AIDS among young women and associated socioeconomic disparities in the most recent years, we followed the DHS guidelines and adjusted for sampling weight, clustering, and stratification variables.^15,16^ For the 40 countries with at least 2 surveys between 2003 and 2018 (eFigure 1 in the Supplement), we estimated the changes in the level of knowledge of HIV/AIDS and associated socioeconomic disparities between the earliest (round 1) and the most recent (round 2) surveys. The period between the 2 selected surveys ranged from 4 to 15 years, with a median (interquartile range) of 9.0 (6.0-10.0) years. The change in the level of knowledge of HIV/AIDS in a country was estimated by calculating the difference between levels at the most recent and earliest surveys.^16^ To measure the change of level of knowledge and its 95% CIs between the 2 survey rounds, we used generalized linear models (GLMs) (binomial distribution and identity link)^22^ with a dichotomous dependent variable indicating a young woman reporting correct knowledge of HIV/AIDS and a dichotomous independent variable indicating survey rounds. To measure the disparity change and its 95% CIs between the 2 survey rounds, we used the GLM (binomial distribution and identity link) with dichotomous knowledge indicator as the dependent variable and 3 independent variables (survey indicator, SES indicator, and an interaction variable between the survey and SES indicators). We then used linear combination to generate the change in disparities between the 2 survey rounds.^16,22^

To generate aggregate-level estimates, we followed previous research and used random-effects meta-analysis by pooling levels and changes of disparity estimates across countries.^21^ This approach does not assume a common homogeneous estimate across the countries with the DerSimonian and Laird inverse-variance method. The DerSimonian-Laird random-effects model combines the survey-specific estimates and weights from random-effects meta-analysis.^21^

Statistical significance was set at *P* < .05, and all tests were 2-tailed. Statistical analyses were performed using Stata version 13 (StataCorp).

## Results

Using the most recent surveys from 51 LMICs after 2010, we included 282 757 young women (166 231 [57.7%] living in rural areas; 56 230 [19.9%] living in households in the lowest wealth quintile; 122 538 [43.3%] with ≤primary education) in the analysis. For the 40 LMICS with at least 2 surveys, we included 398 661 young women (229 231 [57.5%] living in rural areas; 78 148 [19.6%] living in households in the lowest wealth quintile; 181 959 [45.6%] with ≤primary education) in the analysis (eFigure 1 in the Supplement).

### Levels of and Changes in Knowledge About HIV/AIDS

On the aggregate level, using surveys for the most recent years, the level of reported knowledge of HIV/AIDS among the 51 countries was low among young women (29.3%; 95% CI, 24.1%-34.5%) (eTable 2 in the Supplement). On the country level, the level of reported knowledge of HIV/AIDS ranged from 1.0% (95% CI, 0.7%-1.3%) in Afghanistan in 2015 to 64.9% (95% CI, 63.3%-66.5%) in Rwanda in 2014 to 2015 (Figure; eTable 2 in the Supplement).

**Figure.**
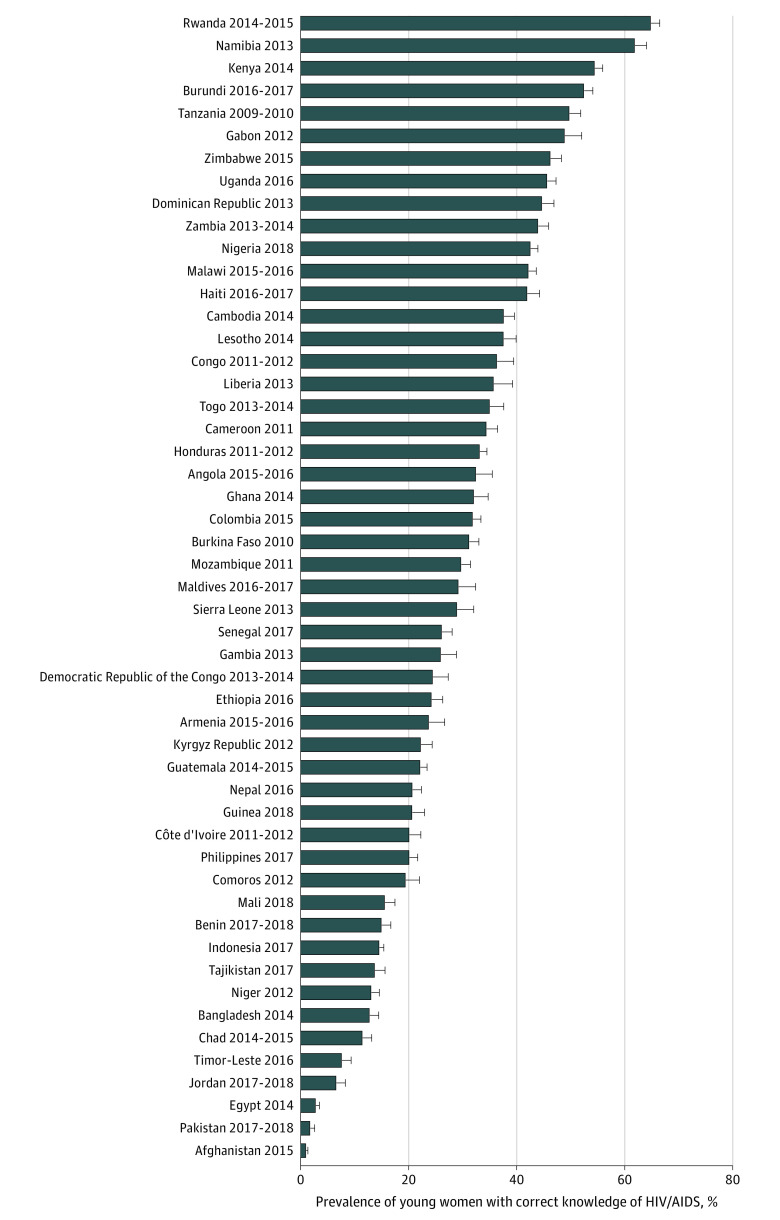
Percentage of Young Women Who Reported Correct Knowledge of HIV/AIDS, Using the Most Recent Surveys Error bars indicate 95% CIs.

For the 40 countries with data in at least 2 survey rounds, the aggregate-level percentage of young women who reported correct knowledge of HIV/AIDS increased significantly, from 26.2% (95% CI, 21.5%-30.9%) in round 1 to 30.2% (95% CI, 24.4%-36.0%) in round 2 (Table 1). On the country level, the percentage of young women with knowledge of HIV/AIDS increased significantly in 24 countries (60.0%). Nigeria, Kenya, and Malawi had the largest increases, at 22.4 (95% CI, 19.4-25.3) percentage points between 2003 and 2018, 15.0 (95% CI, 12.3-17.7) percentage points between 2003 and 2014, and 15.0 (95% CI, 12.9-17.2) percentage points between 2004 to 2005 and 2015 to 2016, respectively. However, a significant reduction was also observed in 10 countries (25.0%; Benin, Cambodia, Egypt, Ghana, Jordan, Maldives, Mali, Nepal, Pakistan, and Timor-Leste) (Table 1).

**Table 1.** Levels of and Changes in Knowledge of HIV/AIDS Among Young Women in 40 Countries With 2 Demographic and Health Surveys^a^

Country	Round 1^b^		Round 2^c^		Difference, percentage points (95% CI)^d^
	Survey years	Level, % (95% CI)	Survey years	Level, % (95% CI)	
All countries^e^	NA	26.2 (21.5 to 30.9)	NA	30.2 (24.4 to 36.0)	4.0 (1.8 to 6.3)
Armenia	2005	22.7 (20.1 to 25.3)	2015-2016	23.8 (20.9 to 26.7)	1.1 (−2.7 to 4.9)
Bangladesh	2007	8.0 (7.0 to 8.9)	2014	12.8 (11.2 to 14.4)	4.9 (3.0 to 6.7)
Benin	2006	23.4 (21.9 to 24.9)	2017-2018	15.0 (13.4 to 16.7)	−8.4 (−10.7 to −6.0)
Burundi	2010-2011	46.3 (44.3 to 48.3)	2016-2017	52.5 (51.0 to 54.1)	6.2 (3.5 to 8.9)
Cambodia	2005-2006	50.1 (48.0 to 52.2)	2014	37.7 (35.7 to 39.6)	−12.4 (−15.4 to −9.4)
Cameroon	2004	30.6 (29.1 to 32.2)	2011	34.4 (32.4 to 36.5)	3.8 (1.2 to 6.4)
Chad	2004	8.1 (6.5 to 9.8)	2014-2015	11.5 (9.8 to 13.2)	3.4 (0.9 to 5.8)
Colombia	2009-2010	24.1 (23.2 to 24.9)	2015-2016	31.9 (30.4 to 33.4)	7.8 (6.1 to 9.6)
Congo	2005	24.9 (22.7 to 27.2)	2011-2012	36.4 (33.3 to 39.4)	11.5 (7.7 to 15.2)
Dominican Republic	2007	41.0 (39.4 to 42.7)	2013	44.7 (42.5 to 46.9)	3.7 (0.9 to 6.4)
Egypt	2005	4.4 (4.4 to 4.4)	2014	2.9 (2.2 to 3.5)	−1.5 (−2.2 to −0.9)
Ethiopia	2005	20.6 (18.8 to 22.3)	2016	24.3 (22.3 to 26.3)	3.7 (1.0 to 6.4)
Ghana	2003	36.8 (34.2 to 39.5)	2014	32.2 (29.6 to 34.7)	−4.7 (−8.4 to −1.0)
Guinea	2005	17.1 (15.2 to 18.9)	2018	20.7 (18.5 to 22.9)	3.6 (0.8 to 6.5)
Haiti	2005-2006	33.9 (31.9 to 35.9)	2016-2017	42.0 (39.7 to 44.2)	8.1 (5.0 to 11.1)
Honduras	2005-2006	29.8 (28.6 to 31.1)	2011-2012	33.2 (31.9 to 34.5)	3.3 (1.5 to 5.2)
Indonesia	2002-2003	1.3 (0.6 to 2.0)	2017	14.6 (13.8 to 15.4)	13.3 (12.3 to 14.4)
Jordan	2007	12.5 (10.5 to 14.6)	2017-2018	6.6 (5.0 to 8.3)	−5.9 (−8.8 to −3.0)
Kenya	2003	39.5 (37.3 to 41.7)	2014	54.5 (53.0 to 55.9)	15.0 (12.3 to 17.7)
Lesotho	2004-2005	27.5 (25.5 to 29.4)	2014	37.6 (35.3 to 39.9)	10.2 (7.2 to 13.2)
Liberia	2006-2007	21.2 (18.9 to 23.5)	2013	35.8 (32.3 to 39.2)	14.5 (10.3 to 18.8)
Malawi	2004-2005	27.2 (25.6 to 28.8)	2015-2016	42.2 (40.8 to 43.6)	15.0 (12.9 to 17.2)
Maldives	2009	33.8 (30.8 to 36.7)	2016-2017	29.3 (26.1 to 32.4)	−4.5 (−8.8 to −0.2)
Mali	2012-2013	23.7 (21.1 to 26.2)	2018	15.6 (13.8 to 17.5)	−8.0 (−11.2 to −4.8)
Mozambique	2003-2004	28.8 (27.0 to 30.7)	2011	29.8 (28.1 to 31.5)	0.9 (−1.6 to 3.4)
Namibia	2006-2007	60.0 (58.0 to 62.0)	2013	61.9 (59.8 to 64.0)	1.9 (−1.0 to 4.8)
Nepal	2005-2006	27.6 (25.4 to 29.9)	2016	20.7 (19.1 to 22.4)	−6.9 (−9.8 to −4.0)
Niger	2006	13.5 (11.9 to 15.0)	2012	13.1 (11.6 to 14.6)	−0.4 (−2.5 to 1.8)
Nigeria	2003	20.3 (17.9 to 22.7)	2018	42.6 (41.3 to 43.9)	22.4 (19.4 to 25.3)
Pakistan	2006-2007	3.4 (2.6 to 4.3)	2017-2018	1.8 (1.0 to 2.6)	−1.6 (−2.8 to −0.5)
Philippines	2008	20.8 (19.3 to 22.2)	2017	20.2 (18.6 to 21.7)	−0.6 (−2.8 to 1.5)
Rwanda	2005	51.1 (49.4 to 52.8)	2014-2015	64.9 (63.3 to 66.5)	13.8 (11.3 to 16.3)
Senegal	2005	19.6 (17.5 to 21.6)	2017	26.2 (24.3 to 28.0)	6.6 (3.8 to 9.4)
Sierra Leone	2008	17.6 (15.4 to 19.7)	2013	29.0 (26.0 to 32.0)	11.4 (7.6 to 15.3)
Tajikistan	2012	9.2 (7.6 to 10.8)	2017	13.8 (11.9 to 15.6)	4.5 (2.0 to 7.1)
Tanzania	2004-2005	45.4 (43.0 to 47.7)	2009-2010	49.8 (47.7 to 51.9)	4.4 (1.1 to 7.7)
Timor-Leste	2009-2010	12.2 (11.0 to 13.4)	2016	7.7 (6.0 to 9.3)	−4.5 (−6.5 to −2.6)
Uganda	2006	32.0 (29.8 to 34.2)	2016	45.7 (44.1 to 47.3)	13.7 (10.8 to 16.5)
Zambia	2007	35.3 (33.2 to 37.4)	2013-2014	44.0 (42.1 to 45.9)	8.7 (5.5 to 11.9)
Zimbabwe	2005-2006	43.4 (41.1 to 45.6)	2015	46.3 (44.4 to 48.3)	3.0 (−0.1 to 6.1)

Abbreviation: NA, not applicable.

^a^ Estimates were weighted and standard errors adjusted for the complex survey design of the Demographic and Health Surveys.

^b^ Round 1 indicates the earliest survey.

^c^ Round 2 indicates the most recent survey.

^d^ Difference indicates absolute change.

^e^ Pooled estimates (ie, aggregate-level estimates) from random-effects meta-analysis, for which the inverse-variance DerSimonian and Laird method was used.

### Residential Area–Associated Disparities and Changes in Knowledge of HIV/AIDS

On the aggregate level, using the data for the most recent years, the level of reported knowledge of HIV/AIDS was 36.3% (95% CI, 30.8%-41.8%) and 23.4% (95% CI, 19.0%-27.9%) for young women in urban and rural areas, respectively, and the gap between the two groups was 12.8 (95% CI, 10.6-14.9) percentage points (eTable 2 in the Supplement). On the country level, the gap between the 2 groups was statistically significant in 46 of 51 LMICs (90.2%), ranging from 2.0 (95% CI, 0.4-3.7) percentage points in Egypt in 2014 to 32.1 (95% CI, 27.9-36.3) percentage points in Angola in 2015 to 2016. This suggested that a larger proportion of young women reported correct knowledge of HIV/AIDS in urban areas in these countries (eFigure 2 and eTable 2 in the Supplement). Only Afghanistan, the Dominican Republic, Jordan, the Philippines, and Tajikistan did not have significant residential area–associated disparities, and knowledge levels in these countries ranged from as low as 1% (Afghanistan, 1.0% [95% CI, 0.7%-1.3%]) to as high as 45% (Dominican Republic, 44.7% [95% CI, 42.5%-46.9%]). There were 9 countries (17.6%) with a gap between the 2 groups of more than 20 percentage points.

For the 40 countries with 2 surveys, the aggregate-level residential area–associated disparities in knowledge of HIV/AIDS did not exhibit a significant decrease between the 2 survey rounds (12.7 [95% CI, 10.4-15.1] percentage points in round 1 vs 11.9 [95% CI, 19.8-14.1] percentage points in round 2) (Table 2). On the country level, only 8 countries (20.0%; Benin, Burundi, Ethiopia, Kenya, Lesotho, Mali, Nepal, and Uganda) showed significant decreases in residential area–associated disparities among young women, either because of an increase in reported knowledge of HIV/AIDS among young women in rural areas (in Burundi, Ethiopia, Kenya, Lesotho, and Uganda) or a decrease in reported knowledge among young women in urban areas (in Benin, Mali, and Nepal). Eight countries (40.0%; Cambodia, Chad, Guinea, Honduras, Indonesia, Namibia, Senegal, and Timor-Leste) showed significant increases in residential area–associated disparities, from 4.6 (95% CI, 1.0-8.2) percentage points in Honduras (from 2005-2006 to 2011-2012) to 7.1 (95% CI, 1.3-13.0) percentage points in Guinea (from 2005 to 2018) (Table 2).

**Table 2.** Level and Changes of Disparities in Knowledge of HIV/AIDS Between Urban and Rural Groups in 40 Countries With 2 Demographic and Health Surveys^a^

Country	Round 1^b^			Round 2^c^			Difference, percentage points (95% CI)^e^
	%		Gap, percentage points (95% CI)^d^	%		Gap, percentage points (95% CI)^d^	
	Urban	Rural		Urban	Rural		
All countries^f^	35.1	21.5	12.7 (10.4 to 15.1)	37.3	25.3	11.9 (9.8 to 14.1)	−0.7 (−2.3 to 0.9)
Armenia	25.8	17.4	8.4 (3.7 to 13.1)	28.7	17.5	11.3 (6.0 to 16.6)	2.9 (−4.1 to 9.8)
Bangladesh	12.9	6.7	6.2 (3.9 to 8.5)	16.7	11.4	5.3 (2.0 to 8.5)	−1.0 (−4.9 to 3.0)
Benin	34.6	14.2	20.4 (17.5 to 23.3)	20.1	11.3	8.8 (5.4 to 12.1)	−11.6 (−16.2 to −7.1)
Burundi	63.6	44.0	19.6 (15.1 to 24.0)	62.1	50.9	11.2 (7.4 to 15.0)	−8.4 (−14.4 to −2.4)
Cambodia	62.1	47.1	15.1 (10.6 to 19.5)	55.0	33.4	21.6 (17.4 to 25.7)	6.5 (0.3 to 12.7)
Cameroon	40.6	16.6	24.0 (21.1 to 26.9)	44.2	21.4	22.8 (18.9 to 26.8)	−1.2 (−6.1 to 3.7)
Chad	17.7	5.2	12.5 (8.3 to 16.7)	25.5	6.2	19.3 (14.9 to 23.6)	6.8 (0.4 to 13.1)
Colombia	26.0	17.1	8.9 (7.0 to 10.7)	34.4	23.5	10.9 (8.2 to 13.6)	2.0 (−1.3 to 5.4)
Congo	27.9	20.9	7.0 (1.9 to 12.1)	39.6	28.1	11.5 (6.1 to 16.9)	4.5 (−2.9 to 11.9)
Dominican Republic	42.7	37.0	5.7 (2.7 to 8.6)	45.0	43.8	1.2 (−4.1 to 6.6)	−4.5 (−10.5 to 1.6)
Egypt	6.0^g^	3.7	2.2 (2.2 to 2.3)	4.4	2.4	2.0 (0.4 to 3.7)	−0.2 (−2.0 to 1.6)
Ethiopia	44.6	14.1	30.5 (26.0 to 35.0)	41.7	18.8	22.8 (18.3 to 27.4)	−7.7 (−14.0 to −1.3)
Ghana	43.7	28.8	14.8 (9.7 to 20.0)	38.5	25.5	13.1 (8.0 to 18.2)	−1.8 (−9.1 to 5.5)
Guinea	23.8	12.8	11.0 (7.2 to 14.9)	30.9	12.7	18.2 (13.7 to 22.6)	7.1 (1.3 to 13.0)
Haiti	41.2	26.7	14.5 (10.4 to 18.6)	47.2	37.5	9.7 (5.1 to 14.3)	−4.8 (−11.0 to 1.4)
Honduras	36.8	21.7	15.0 (12.6 to 17.5)	42.2	22.6	19.7 (17.1 to 22.2)	4.6 (1.0 to 8.2)
Indonesia	1.4	1.2	0.2 (−1.0 to 1.4)	17.6	11.2	6.4 (4.8 to 8.0)	6.2 (4.2 to 8.2)
Jordan	13.2	8.8	4.4 (1.0 to 7.8)	6.6	6.8	−0.2 (−4.0 to 3.6)	−4.6 (−9.8 to 0.7)
Kenya	52.3	34.8	17.5 (12.9 to 22.0)	60.8	50.3	10.5 (7.5 to 13.6)	−6.9 (−12.5 to −1.3)
Lesotho	42.6	23.4	19.3 (14.6 to 23.9)	43.8	34.5	9.3 (4.3 to 14.3)	−9.9 (−16.8 to −3.1)
Liberia	27.0	15.6	11.4 (6.7 to 16.1)	40.0	27.4	12.6 (6.8 to 18.4)	1.2 (−6.4 to 8.8)
Malawi	32.5	25.8	6.6 (2.0 to 11.2)	48.7	40.8	7.9 (3.7 to 12.1)	1.2 (−5.1 to 7.6)
Maldives	43.5	30.1	13.4 (5.1 to 21.7)	37.0	21.5	15.5 (9.3 to 21.8)	2.1 (−8.2 to 12.5)
Mali	35.7	18.6	17.1 (11.9 to 22.3)	20.8	13.5	7.3 (2.6 to 11.9)	−9.9 (−16.9 to −2.8)
Mozambique	38.0	19.8	18.1 (14.5 to 21.7)	39.5	23.9	15.6 (12.3 to 18.9)	−2.5 (−7.4 to 2.4)
Namibia	63.5	57.2	6.3 (2.2 to 10.5)	67.7	55.1	12.6 (8.4 to 16.8)	6.3 (0.4 to 12.1)
Nepal	42.6	24.8	17.8 (12.4 to 23.2)	24.7	14.3	10.4 (7.1 to 13.8)	−7.4 (−13.9 to −0.9)
Niger	31.5	8.1	23.4 (19.7 to 27.1)	30.0	8.6	21.4 (17.5 to 25.3)	−2.0 (−7.3 to 3.4)
Nigeria	29.1	15.2	13.9 (8.7 to 19.1)	51.4	35.7	15.6 (12.8 to 18.5)	1.8 (−4.3 to 7.9)
Pakistan	5.1	2.8	2.4 (0.3 to 4.5)	3.5	1.1	2.4 (0.4 to 4.4)	−0.0 (−2.9 to 2.9)
Philippines	23.5	17.2	6.3 (3.5 to 9.0)	21.5	18.8	2.7 (−0.5 to 5.9)	−3.6 (−7.8 to 0.7)
Rwanda	63.3	48.3	15.0 (11.3 to 18.7)	73.8	62.5	11.3 (7.8 to 14.7)	−3.7 (−9.1 to 1.7)
Senegal	27.8	11.6	16.3 (12.7 to 19.8)	38.1	14.9	23.2 (19.8 to 26.7)	7.0 (2.0 to 11.9)
Sierra Leone	28.4	9.0	19.4 (15.0 to 23.8)	38.5	22.3	16.2 (10.0 to 22.4)	−3.2 (−10.9 to 4.5)
Tajikistan	11.8	8.5	3.3 (−0.1 to 6.8)	16.1	13.1	3.0 (−0.2 to 6.3)	−0.3 (−4.9 to 4.3)
Tanzania	57.1	40.4	16.7 (11.8 to 21.6)	57.0	46.5	10.5 (6.1 to 14.8)	−6.2 (−13.1 to 0.6)
Timor-Leste	14.0	11.6	2.5 (−0.3 to 5.3)	12.6	4.8	7.8 (3.8 to 11.7)	5.3 (0.9 to 9.7)
Uganda	48.3	28.1	20.3 (14.2 to 26.3)	54.7	42.4	12.4 (8.8 to 15.9)	−7.9 (−15.1 to −0.7)
Zambia	43.6	28.2	15.4 (11.1 to 19.8)	54.8	33.7	21.1 (17.5 to 24.8)	5.7 (−0.1 to 11.5)
Zimbabwe	50.2	38.5	11.7 (7.4 to 16.0)	55.7	40.8	14.9 (11.0 to 18.9)	3.2 (−2.8 to 9.3)

^a^ Estimates were weighted and standard errors adjusted for the complex survey design of the Demographic and Health Surveys.

^b^ Round 1 indicates the earliest survey.

^c^ Round 2 indicates the most recent survey.

^d^ Gap indicates the absolute difference between the 2 subgroups.

^e^ Difference indicates absolute change in gaps between rounds.

^f^ Pooled estimates (ie, aggregate-level estimates) from random-effects meta-analysis, for which the inverse-variance DerSimonian and Laird method was used.

^g^ Missing standard errors because of stratum with single sampling unit (not included in the pooled analysis).

### Wealth-Associated Disparities and Changes in Knowledge of HIV/AIDS

On the aggregate level, the level of knowledge of HIV/AIDS in the 51 countries with the most recent surveys was 40.5% (95% CI, 34.5%-46.5%) and 18.6% (95% CI, 15.6%-21.5%) among young women in the highest and lowest wealth quintiles. The gap between the 2 groups was 21.8 (95% CI, 18.3-25.3) percentage points (eTable 2 in the Supplement). On the country level, the gap between the richest and the poorest groups was statistically significant in 50 LMICs (98.0%), ranging from 1.4 (95% CI, 0.0-2.8) percentage points in Afghanistan in 2015 to 49.6 (95% CI, 43.8-55.4) percentage points in Angola in 2015 to 2016 (eFigure 3 and eTable 2 in the Supplement). There were 29 countries (56.8%) with a gap between the two groups of more than 20 percentage points.

Similar to residential area–associated disparities, for the 40 countries with data in 2 survey rounds, the aggregate wealth-associated disparities in reported knowledge of HIV/AIDS did not exhibit a significant decrease (20.9 [95% CI, 17.6-24.2] percentage points in round 1 vs 21.1 [95% CI, 17.9-24.4] percentage points in round 2) (Table 3). On the country level, only 5 of 40 countries (12.5%; Benin, Burundi, Cambodia, Egypt, and Nepal) showed a significant decrease in wealth-associated disparities either because of an increase in reported knowledge of HIV/AIDS among the group in the lowest quintile (Burundi) or a decrease in reported knowledge among the group in the highest quintile (Benin, Cambodia, Egypt and Nepal). Six countries (15.0%; Congo, Guinea, Honduras, Indonesia, Namibia, and Senegal) showed significant increases in wealth-associated disparities (Table 3).

**Table 3.** Level and Changes of Disparities in Knowledge of HIV/AIDS Between the Highest and Lowest Wealth Quintiles in the 40 Countries With 2 Demographic and Health Surveys^a^

Country	Round 1^b^			Round 2^c^			Difference, percentage points (95% CI)^e^
	%		Gap, percentage points (95% CI)^d^	%		Gap, percentage points (95% CI)^d^	
	Highest	Lowest		Highest	Lowest		
All countries^f^	38.1	14.0	20.9 (17.6 to 24.1)	41.0	19.8	21.1 (17.9 to 24.4)	0.3 (−2.1 to 2.7)
Armenia	29.4	12.0	17.4 (11.9 to 22.9)	34.4	15.0	19.4 (9.5 to 29.2)	1.9 (−10.0 to 13.9)
Bangladesh	14.8	2.1	12.7 (9.8 to 15.6)	21.2	7.7	13.5 (7.5 to 19.4)	0.7 (−5.9 to 7.4)
Benin	41.4	10.9	30.6 (26.9 to 34.2)	23.6	7.3	16.3 (11.9 to 20.6)	−14.3 (−20.0 to −8.5)
Bolivia	31.4	2.1	29.3 (26.4 to 32.2)	37.7	3.4	34.2 (31.0 to 37.4)	4.9 (0.6 to 9.3)
Burundi	62.7	35.6	27.1 (21.6 to 32.6)	59.5	44.8	14.7 (10.1 to 19.3)	−12.4 (−19.7 to −5.1)
Cambodia	68.4	26.1	42.3 (37.4 to 47.2)	54.9	26.6	28.3 (23.1 to 33.6)	−14.0 (−21.2 to −6.8)
Cameroon	50.7	10.5	40.2 (36.4 to 44.0)	52.1	8.0	44.1 (39.0 to 49.2)	3.9 (−2.5 to 10.3)
Chad	17.7	0.9	16.8 (12.5 to 21.1)	25.4	6.5	19.0 (13.8 to 24.1)	2.2 (−4.8 to 9.1)
Colombia	32.0	14.8	17.3 (14.4 to 20.1)	41.2	22.1	19.0 (13.0 to 25.1)	1.8 (−4.9 to 8.5)
Congo	34.9	18.5	16.4 (9.6 to 23.2)	49.8	23.3	26.5 (19.3 to 33.7)	10.1 (0.1 to 20.1)
Dominican Republic	46.7	31.2	15.5 (10.2 to 20.7)	51.7	33.5	18.2 (10.4 to 26.1)	2.7 (−6.7 to 12.2)
Egypt	10.3^g^	0.9	9.4 (9.3 to 9.5)	5.0	0.7	4.4 (2.0 to 6.7)	−5.0 (−7.8 to −2.2)
Ethiopia	38.7	8.3	30.4 (26.2 to 34.7)	39.7	11.5	28.1 (23.3 to 32.9)	−2.3 (−8.7 to 4.1)
Ghana	48.5	26.3	22.1 (15.5 to 28.8)	43.0	14.3	28.7 (22.5 to 34.9)	6.6 (−2.5 to 15.6)
Guinea	27.0	9.6	17.4 (11.7 to 23.1)	35.3	7.6	27.6 (22.7 to 32.6)	10.3 (2.7 to 17.8)
Haiti	43.9	19.7	24.2 (18.8 to 29.6)	51.0	30.0	21.0 (14.9 to 27.1)	−3.2 (−11.3 to 4.9)
Honduras	42.7	12.7	30.1 (26.5 to 33.6)	47.7	12.0	35.7 (32.3 to 39.1)	5.6 (0.7 to 10.5)
Indonesia	3.1	0.2	2.9 (−0.0 to 5.8)	21.7	7.2	14.5 (12.1 to 16.9)	11.6 (7.8 to 15.4)
Jordan	26.9	9.6	17.4 (4.5 to 30.3)	12.5	5.8	6.7 (−3.2 to 16.7)	−10.6 (−28.0 to 6.7)
Kenya	52.0	25.2	26.8 (20.3 to 33.3)	63.1	35.0	28.1 (23.7 to 32.5)	1.3 (−6.6 to 9.2)
Lesotho	40.8	14.4	26.4 (20.4 to 32.4)	47.6	26.4	21.2 (13.9 to 28.5)	−5.2 (−14.6 to 4.2)
Liberia	30.2	14.4	15.8 (7.9 to 23.6)	46.4	21.6	24.8 (17.3 to 32.2)	9.0 (−1.9 to 19.9)
Malawi	34.3	21.0	13.2 (8.6 to 17.9)	49.9	35.5	14.4 (10.1 to 18.7)	1.2 (−5.2 to 7.6)
Maldives	46.1	22.8	23.3 (12.3 to 34.4)	40.9	18.4	22.4 (12.8 to 32.0)	−0.9 (−15.5 to 13.7)
Mali	36.2	14.8	21.3 (15.3 to 27.4)	24.0	9.0	15.0 (9.7 to 20.3)	−6.3 (−14.4 to 1.7)
Mozambique	40.4	15.4	25.0 (19.6 to 30.4)	41.9	20.9	21.0 (16.4 to 25.6)	−4.0 (−11.1 to 3.1)
Namibia	70.2	55.8	14.4 (8.3 to 20.4)	71.5	48.0	23.5 (17.5 to 29.5)	9.2 (0.7 to 17.7)
Nepal	49.3	11.6	37.7 (32.1 to 43.4)	38.3	14.0	24.4 (19.2 to 29.5)	−13.4 (−20.9 to −5.8)
Niger	30.5	4.6	25.8 (22.0 to 29.7)	28.9	5.0	23.9 (20.1 to 27.7)	−1.9 (−7.3 to 3.5)
Nigeria	33.0	11.6	21.4 (15.3 to 27.5)	56.3	30.1	26.2 (22.2 to 30.3)	4.8 (−2.7 to 12.4)
Pakistan	8.6	0.1	8.5 (5.1 to 11.9)	5.1	0.3	4.8 (1.6 to 7.9)	−3.7 (−8.3 to 0.9)
Peru	30.6	2.7	27.9 (24.8 to 31.0)	34.7	5.6	29.1 (24.8 to 33.4)	1.2 (−4.1 to 6.5)
Philippines	26.4	14.4	12.0 (8.2 to 15.8)	25.3	11.6	13.7 (9.1 to 18.3)	1.6 (−4.4 to 7.7)
Rwanda	58.9	42.5	16.4 (11.4 to 21.4)	71.5	57.5	14.0 (9.2 to 18.7)	−2.4 (−9.3 to 4.6)
Senegal	32.6	9.0	23.6 (18.3 to 28.9)	49.6	5.7	43.9 (39.2 to 48.6)	20.3 (13.2 to 27.3)
Sierra Leone	31.2	6.0	25.2 (20.1 to 30.3)	40.9	21.4	19.5 (11.9 to 27.2)	−5.7 (−14.9 to 3.6)
Tajikistan	11.4	7.0	4.3 (−0.2 to 8.8)	19.4	10.0	9.4 (4.9 to 13.9)	5.1 (−1.0 to 11.2)
Tanzania	57.7	34.7	23.0 (16.3 to 29.7)	57.1	40.1	17.0 (10.0 to 24.1)	−5.9 (−15.8 to 4.0)
Timor-Leste	15.8	8.9	6.9 (3.1 to 10.6)	14.8	2.5	12.4 (7.4 to 17.3)	5.5 (−0.1 to 11.1)
Uganda	47.3	20.3	27.0 (21.6 to 32.4)	57.6	33.9	23.7 (19.8 to 27.7)	−3.3 (−10.1 to 3.5)
Zambia	49.9	23.3	26.5 (20.9 to 32.1)	60.8	29.3	31.5 (26.6 to 36.3)	4.9 (−2.6 to 12.5)
Zimbabwe	52.3	30.6	21.7 (17.1 to 26.3)	57.9	34.4	23.5 (17.4 to 29.6)	1.8 (−6.0 to 9.6)

^a^ Estimates were weighted and standard errors adjusted for the complex survey design of the Demographic and Health Surveys.

^b^ Round 1 indicates the earliest survey.

^c^ Round 2 indicates the most recent survey.

^d^ Gap indicates the absolute difference between the 2 subgroups.

^e^ Difference indicates absolute change in gaps between rounds.

^f^ Pooled estimates (ie, aggregate-level estimates) from random-effects meta-analysis, for which the inverse-variance DerSimonian and Laird method was used.

^g^ Missing standard errors because of stratum with single sampling unit (not included in the pooled analysis).

### Education-Associated Disparities and Changes in Knowledge of HIV/AIDS

On the aggregate level, reported knowledge of HIV/AIDS was 37.8% (95% CI, 31.9%-43.6%) and 18.4% (95% CI, 15.1%-21.8%) between young women with education higher than primary and those with primary education or less, respectively. The gap between the 2 groups was 19.4 (95% CI, 16.6-22.2) percentage points (eTable 2 in the Supplement). On the country level, the gap between the 2 groups was significant in 50 of 51 LMICs (98.0%), ranging from 1.8 (95% CI, 0.7-2.9) percentage points in Afghanistan in 2015 to 38.9 (95% CI, 35.2-42.7) percentage points in Burkina Faso in 2010 (eFigure 4 and eTable 2 in the Supplement). There were 24 countries (47.1%) with a gap between the 2 groups of more than 20 percentage points.

For the 40 countries with data in 2 survey rounds, education-associated disparities in reported knowledge of HIV/AIDS exhibited a significant decrease on the aggregate level, from 21.6 (95% CI, 17.7-25.4) percentage points in round 1 to 18.5 (96% CI, 15.6-21.3) percentage points in round 2 (Table 4). On the country level, 17 LMICs (42.5%) showed a significant decrease in education-associated disparities because of an increase in reported knowledge of HIV/AIDS among young women with primary education or less (Burundi, Ethiopia, Haiti, Lesotho, Rwanda, Sierra Leone, and Uganda) or a decrease in knowledge among young women with education greater than primary (Benin, Cambodia, Egypt, Mali, Mozambique, Nepal, Niger, Pakistan, Senegal, and Timor-Leste). Six countries (15.0%) showed a significant increase in education-associated disparities (Indonesia, Liberia, Malawi, Namibia, Nigeria, and Tajikistan) (Table 4).

**Table 4.** Level and Changes of Disparities in Knowledge of HIV/AIDS Between the Group With Greater Than Primary Education and the Group With Primary Education or Less in the 40 Countries With 2 Demographic and Health Surveys^a^

Country	Round 1^b^			Round 2^c^			Difference, percentage points (95% CI)^e^
	%		Gap, percentage points (95% CI)^d^	%		Gap, percentage points (95% CI)^d^	
	>Primary education	≤Primary education		>Primary education	≤Primary education		
All countries^f^	39.2	17.9	21.6 (17.7 to 25.4)	38.1	19.6	18.5 (15.6 to 21.3)	−3.2 (−5.4 to −1.0)
Armenia	22.7	40.9^g^	−18.2 (−61.2 to 24.7)	25.4	8.9	16.5 (11.2 to 21.8)	34.7 (−8.4 to 77.8)
Bangladesh	12.1	2.7	9.4 (7.3 to 11.5)	16.7	5.6	11.1 (9.2 to 13.1)	1.8 (−1.1 to 4.6)
Benin	48.8	13.5	35.3 (32.3 to 38.3)	24.7	8.5	16.2 (13.3 to 19.2)	−19.1 (−23.4 to −14.8)
Burundi	70.9	41.1	29.9 (26.1 to 33.6)	62.9	44.9	18.0 (15.2 to 20.8)	−11.8 (−16.5 to −7.2)
Cambodia	72.9	37.2	35.7 (32.4 to 39.0)	45.9	23.8	22.1 (18.9 to 25.3)	−13.6 (−18.2 to −9.0)
Cameroon	50.5	13.1	37.4 (34.6 to 40.2)	48.1	14.4	33.7 (30.4 to 37.1)	−3.7 (−8.0 to 0.6)
Chad	22.3	6.5	15.8 (9.7 to 21.9)	25.1	7.1	18.0 (13.2 to 22.9)	2.2 (−5.6 to 10.0)
Colombia	25.8	9.0	16.8 (14.9 to 18.7)	33.2	13.6	19.6 (16.4 to 22.8)	2.8 (−0.9 to 6.5)
Congo	32.8	13.3	19.5 (16.0 to 23.0)	41.1	22.9	18.3 (12.7 to 23.9)	−1.2 (−7.8 to 5.4)
Dominican Republic	46.1	29.3	16.8 (13.8 to 19.8)	48.4	31.7	16.6 (11.5 to 21.8)	−0.2 (−6.1 to 5.8)
Egypt	6.5	1.3	5.2 (5.2 to 5.2)	3.4	0.4	3.1 (2.1 to 4.0)	−2.1 (−3.1 to −1.1)
Ethiopia	52.0	14.2	37.7 (32.8 to 42.7)	42.8	17.9	25.0 (20.7 to 29.2)	−12.8 (−19.3 to −6.3)
Ghana	45.6	22.3	23.3 (18.5 to 28.1)	39.0	13.1	25.9 (22.1 to 29.8)	2.6 (−3.5 to 8.7)
Guinea	38.6	12.0	26.7 (22.8 to 30.6)	37.4	13.4	24.0 (19.7 to 28.3)	−2.7 (−8.4 to 3.1)
Haiti	46.1	23.4	22.7 (19.0 to 26.4)	47.2	30.5	16.8 (13.0 to 20.6)	−5.9 (−11.2 to −0.6)
Honduras	43.3	18.7	24.6 (22.3 to 26.9)	43.7	16.9	26.9 (24.6 to 29.1)	2.2 (−1.0 to 5.5)
Indonesia	2.3	0.2	2.1 (0.8 to 3.4)	15.6	4.6	10.9 (9.0 to 12.9)	8.8 (6.5 to 11.1)
Jordan	12.5	14.0	−1.5 (−15.4 to 12.3)	7.2	0.9	6.3 (4.3 to 8.3)	7.8 (−6.2 to 21.8)
Kenya	54.2	32.8	21.5 (17.1 to 25.8)	65.2	43.5	21.7 (19.1 to 24.2)	0.2 (−4.8 to 5.3)
Lesotho	42.1	16.5	25.6 (21.4 to 29.8)	42.8	24.1	18.7 (14.3 to 23.1)	−6.9 (−13.0 to −0.9)
Liberia	32.7	16.6	16.1 (11.2 to 21.1)	49.4	25.6	23.8 (18.4 to 29.2)	7.7 (0.3 to 15.0)
Malawi	36.9	24.3	12.6 (8.9 to 16.3)	55.3	36.4	19.0 (16.1 to 21.8)	6.4 (1.7 to 11.0)
Maldives	36.9	17.9	19.0 (13.0 to 25.0)	29.5	12.2	17.3 (7.8 to 26.8)	−1.7 (−12.9 to 9.6)
Mali	40.1	17.5	22.6 (17.6 to 27.6)	25.2	10.7	14.4 (10.9 to 18.0)	−8.1 (−14.3 to −2.0)
Mozambique	54.0	23.9	30.1 (25.4 to 34.8)	44.7	23.9	20.8 (17.2 to 24.5)	−9.3 (−15.2 to −3.4)
Namibia	65.7	41.0	24.7 (20.8 to 28.7)	67.8	37.0	30.8 (26.7 to 35.0)	6.1 (0.3 to 11.8)
Nepal	47.8	8.8	39.0 (36.0 to 42.0)	25.8	4.8	21.0 (18.6 to 23.4)	−18.0 (−21.8 to −14.2)
Niger	48.7	9.9	38.8 (33.3 to 44.3)	35.0	9.3	25.7 (21.2 to 30.1)	−13.1 (−20.1 to −6.1)
Nigeria	26.2	13.7	12.5 (8.1 to 16.8)	49.6	31.7	17.9 (15.2 to 20.6)	5.5 (0.2 to 10.7)
Pakistan	11.6	1.1	10.5 (7.5 to 13.4)	4.6	0.3	4.3 (2.2 to 6.4)	−6.2 (−9.8 to −2.5)
Philippines	22.5	9.0	13.5 (11.0 to 16.0)	21.0	9.9	11.0 (7.7 to 14.4)	−2.5 (−6.8 to 1.8)
Rwanda	70.2	49.3	21.0 (16.5 to 25.4)	70.7	61.1	9.6 (6.8 to 12.4)	−11.4 (−16.8 to −5.9)
Senegal	52.8	12.2	40.6 (36.5 to 44.6)	41.4	11.8	29.6 (26.4 to 32.8)	−11.0 (−16.1 to −5.8)
Sierra Leone	35.6	8.4	27.2 (23.0 to 31.4)	39.1	17.5	21.6 (18.0 to 25.1)	−5.7 (−11.2 to −0.1)
Tajikistan	9.7	4.1	5.6 (2.5 to 8.7)	14.3	1.0	13.3 (11.1 to 15.5)	7.7 (4.0 to 11.4)
Tanzania	65.2	43.0	22.2 (16.3 to 28.1)	61.8	45.3	16.5 (11.8 to 21.2)	−5.7 (−13.6 to 2.2)
Timor-Leste	16.3	4.1	12.2 (10.6 to 13.9)	8.8	2.8	6.1 (3.1 to 9.1)	−6.2 (−9.4 to −2.9)
Uganda	52.4	23.5	28.8 (25.0 to 32.6)	59.9	36.5	23.4 (20.7 to 26.1)	−5.4 (−10.2 to −0.7)
Zambia	49.1	24.3	24.8 (20.8 to 28.8)	53.1	30.4	22.7 (19.1 to 26.4)	−2.1 (−7.4 to 3.3)
Zimbabwe	49.0	27.9	21.1 (17.7 to 24.6)	51.4	28.5	22.9 (18.7 to 27.0)	1.8 (−3.6 to 7.1)

^a^ Estimates were weighted and standard errors adjusted for the complex survey design of the Demographic and Health Surveys.

^b^ Round 1 indicates the earliest survey.

^c^ Round 2 indicates the most recent survey.

^d^ Gap indicates the absolute difference between the 2 subgroups.

^e^ Difference indicates the absolute change in gaps between rounds.

^f^ Pooled estimates (ie, aggregate-level estimates) from random-effects meta-analysis, for which the inverse-variance DerSimonian and Laird method was used.

^g^ Missing standard errors because of stratum with single sampling unit (not included in the pooled analysis).

## Discussion

To our knowledge, this is the first study to systematically assess the level of and changes in socioeconomic disparities in reported knowledge of HIV/AIDS among young women aged 15 to 24 years in 51 LMICs using the most up-to-date data. We found that although reported knowledge of HIV/AIDS has increased over time on the aggregate level, it was still low; less than one-third of young women reported correct knowledge of HIV/AIDS. The study also revealed that in most of the countries studied, residential area–associated, education-associated, and wealth-associated disparities in knowledge remained large. In some countries, the disparities even increased significantly, as the knowledge of the population with higher SES improved more than its lower-SES counterpart.

Our results regarding the low level of knowledge of HIV/AIDS are consistent with previous studies.^5,8,9^ Moreover, the finding that socioeconomic disparities existed in almost all countries studied is consistent with previous findings about disparities in health information transmission: people with higher SES have better access to health information regarding HIV/AIDS and better access to health consultation than people with lower SES.^4,5,23,24^ It is worth noting that in many LMICs, disparities did not decline as the level of knowledge of HIV/AIDS improved. The presence of large socioeconomic disparities raises serious concerns about the effectiveness of combating HIV/AIDS when young women with low SES are left behind. Meanwhile, in some countries, the disparities in knowledge narrowed because of a decrease in the percentage of young women who reported correct knowledge in the group with high SES. These findings highlight the need to provide education on HIV/AIDS to all young women, with additional support for those with low SES, especially in countries with low overall levels of knowledge.

Knowledge of HIV/AIDS is an important determinant for successful HIV prevention and control of transmission.^4,6,7^ Inadequate knowledge is associated with lower use of HIV-prevention services (eg, condoms), more behaviors that put one’s health at risk (eg, unsafe sexual behaviors),^11,13,25,26,27^ and a higher risk of HIV infection and/or transmission.^24,25,26,27,28^ The findings of this study clearly indicate that more efforts are needed to improve the knowledge of HIV/AIDS among young women with low SES. Studies have discovered effective methods for raising awareness of and delivering correct knowledge about HIV/AIDS among young people. School-based HIV/AIDS education is a well-proven intervention strategy to enhance young people’s knowledge of HIV/AIDS^10^ that has been implemented in many LMICs.^29^ We need to continue this intervention strategy to increase knowledge of HIV among young populations. Meanwhile, in many LMICs, more and more young people preferred to access HIV/AIDS information through mass media (eg, radio, television, newspapers) or social media (eg, mobile applications or social networking websites) than through medical professionals or family members.^4,5,23,27,28^ The use of social media has been proven to be complementary to school-based education and to extend the dissemination of knowledge about HIV/AIDS among young people.^30^ In some countries, mass and social media are the primary sources of HIV/AIDS information among young people.^23,27,28,30^ Social or mass media could improve the reach of HIV/AIDS information to young people with low SES and provide an important avenue for generating, sharing, and receiving information about HIV.^30^

### Limitations

This study has several limitations. First, the measures of knowledge of HIV/AIDS used may be biased because they were entirely self-reported, but given that all surveys have a similar design, any potential bias should be consistent across countries and survey years. Second, because of unavailable longitudinal data, we had to use repeated cross-sectional data sets from the most recent and earliest DHS surveys to estimate changes.^16^ We need to be cautious when making cross-country comparisons because DHS data from different countries may have been gathered in different years, and there may have been intervals of different lengths between the surveys.^20^ Third, although this is, to our knowledge, the most comprehensive report to date, we were only able to include 51 LMICs in the analysis for the most recent surveys and 40 LMICS in the change analysis. Therefore, our results are not representative on global or regional levels.

## Conclusions

The ongoing inadequacy of knowledge and high socioeconomic disparities among young women in the 51 LMICs studied here is of greatest concern for the prevention and management of HIV/AIDS. Future HIV-prevention campaigns or programs should continue to emphasize the needs of young people with socioeconomic disadvantage and to provide more support for effective interventions, such as public campaigns through mass or social media. Meanwhile, the findings that the gap in knowledge of HIV has largely been narrowed in some countries, such as Burundi, calls for more in-depth studies of how those countries have been able to effectively deliver knowledge of HIV/AIDS to young people with socioeconomic disadvantage. Future research linking our findings to health education policies on HIV/AIDS in these specific countries would be particularly valuable.

## Notes

Supplement. **eAppendix. **Data Sources **eFigure 1. **Flow Diagram of Selecting the DHS With HIV/AIDS Knowledge Variables Between 2000 and 2018 **eFigure 2. **Disparities in Knowledge of HIV/AIDS by Residential Area Using the Most Recent Surveys **eFigure 3. **Disparities in Knowledge of HIV/AIDS by Wealth Quintile Using the Most Recent Surveys **eFigure 4. **Disparities in Knowledge of HIV/AIDS by Education Using the Most Recent Surveys **eTable 1. **Sample Size and Missing Values in 51 LMICs **eTable 2. **Knowledge Level of HIV/AIDS and Associated Socioeconomic Disparities Among Young Women in 51 LIMCs in the Most Recent Survey
