# Error in Byline

**DOI:** 10.1001/jamanetworkopen.2021.3317

**Published:** 2021-02-26

In the Original Investigation titled “Assessment of Knowledge of HIV/AIDS and Association With Socioeconomic Disparities Among Young Women in Low- and Middle-Income Countries, 2003 to 2018,”^1^ published January 22, 2021, there was an error in the byline. The third author’s name should have read S. V. Subramanian. This article has been corrected.^1^
