# Supplementary material for: Assessment of Knowledge of HIV/AIDS and Association With Socioeconomic Disparities Among Young Women in Low- and Middle-Income Countries, 2003 to 2018
Source: JAMA Netw Open. 2021 Jan 22;4(1):e2035000. doi: 10.1001/jamanetworkopen.2020.35000 (PMC7823222; doi:10.1001/jamanetworkopen.2020.35000)
Supplement: Supplement. — eAppendix. Data Sources eFigure 1. Flow Diagram of Selecting the DHS With HIV/AIDS Knowledge Variables Between 2000 and 2018 eFigure 2. Disparities in Knowledge of HIV/AIDS by Residential Area Using the Most Recent Surveys eFigure 3. Disparities in Knowledge of HIV/AIDS by Wealth Quintile Using the Most Recent Surveys eFigure 4. Disparities in Knowledge of HIV/AIDS by Education Using the Most Recent Surveys eTable 1. Sample Size and Missing Values in 51 LMICs eTable 2. Knowledge Level of HIV/AIDS and Associated Socioeconomic Disparities Among Young Women in 51 LIMCs in the Most Recent Survey [file jamanetwopen-e2035000-s001.pdf]

Supplemental Online Content

Yang F, Li Z, Subramianian SV, Lu C. Assessment of knowledge of HIV/AIDS and association with socioeconomic disparities among young women in low- and middle-income countries, 2003 to 2018. *JAMA Netw Open*. 2021;4(1):e2035000. doi:10.1001/jamanetworkopen.2020.35000

eAppendix. Data Sources

eFigure 1. Flow Diagram of Selecting the DHS With HIV/AIDS Knowledge Variables Between 2000 and 2018

eFigure 2. Disparities in Knowledge of HIV/AIDS by Residential Area Using the Most Recent Surveys

eFigure 3. Disparities in Knowledge of HIV/AIDS by Wealth Quintile Using the Most Recent Surveys

eFigure 4. Disparities in Knowledge of HIV/AIDS by Education Using the Most Recent Surveys

eTable 1. Sample Size and Missing Values in 51 LMICs

eTable 2. Knowledge Level of HIV/AIDS and Associated Socioeconomic Disparities Among Young Women in 51 LIMCs in the Most Recent Survey

This supplemental material has been provided by the authors to give readers additional information about their work.

© 2021 Yang F et al. *JAMA Network Open.*

eAppendix. Data Sources

DHS Survey Methods

Demographic and health surveys (DHS) is an ongoing collaboration that was initiated in 1984 between the United States Agency for International Development and country-specific development agencies in 85 low- and middle-income countries (LMICs).[1–3] The DHS were initially designed to expand on demographic, fertility, and family -planning data, and they continue to provide an important resource for the monitoring of vital statistics and population health indicators in LMICs, focusing on maternal and child health, nutrition, reproductive health, fertility and mortality, and adult health behaviors.[1–3] In many countries, the standard DHS is conducted every three to six years.

The analysis of these datasets was conducted using women's surveys from all DHS. A multistage stratified sampling design was used to draw the sample from each DHS survey of the countries. In the first stage, each country was divided into sub-national regions according to political regions (such as state or province) or geographic region (north, south, east and west), and then each sub-region was stratified into urban and rural areas. Within each stratified area, enumeration areas were randomly selected from the most recent population census as the primary sampling unit (clusters), and the probability of extraction per cluster was equal to the proportion of the population of that particular cluster, thus accounting for the total population. In the second stage, all households within each cluster were listed, and then an average of 25 households

© 2021 Yang F et al. *JAMA Network Open.*

within each cluster were drawn randomly using equal probability systematic sampling. Trained interviewers visited these selected households, and completed the household roster after a brief interview with the household, and then identified eligible women aged 15–49 within each household to conduct an individual interview in the women's survey module (some countries have extended the age range of women surveyed, such as 13–49 years in the 2015 Colombia survey and 15–64 years in the 2013 Namibia survey.).

To ensure standardization and comparability across different sites and time, DHS adopted extensive interviewer training, an identical core questionnaire and standardized measurement instruments and technologies. The response rate was higher than 90%.[1–3]

Study Population and Sample Size

The DHS is a national household survey conducted in 85 LMICs. At least two surveys have been conducted in 57 of these countries.[1] The multiple surveys in many countries allow for repeated cross-sectional analyses to systematically identify trends of populations and health in LMICs,[1] such as knowledge of HIV/AIDS. Therefore, to construct large-scale datasets with sufficient statistical capability to monitor changes in the level of knowledge of HIV/AIDS and their relationship to socioeconomic disparities in LMICs, the cross-sectional and repeated cross-sectional datasets were

© 2021 Yang F et al. *JAMA Network Open.*

constructed from women's surveys from all DHS before 2019. To obtain a comprehensive up-to-date assessment on both the country- and aggregate-levels, we selected the countries with the most recent surveys conducted since 2010. All analysis was limited to young women aged 15–24 (**eFigure 1**).

We used the most recent cross-sectional surveys from the countries that had collected information on knowledge of HIV/AIDS and socio-demographic characteristics among young women. In the DHS, 25 countries did not have information related to knowledge of HIV/AIDS, and nine countries had information related to knowledge of HIV/AIDS but their surveys were conducted before 2010. Those 34 countries were excluded from this analysis. The total sample included 51 countries, including 40 countries between 2013 and 2018, and 11 between 2010 to 2012. After the exclusion of 529,056 women aged <15 or >24 and 17,441 with missing data, this analytic dataset contained 282,757 young women aged 15–24.

The change dataset was constructed using repeated cross-sectional surveys for 40 countries that collected information on knowledge of HIV and socio-demographic characteristics among young women in at least two surveys. For the 40 countries, data from the most recent and the earliest surveys were selected for comparison. The average interval between the most recent and the earliest surveys was 8.3 years (standard deviation, 2.8). The longest interval was 15 years for Nigeria (2003–2018), and the shortest interval was 4 years for Tanzania (2004/2005–2009/2010). After the

© 2021 Yang F et al. *JAMA Network Open.*

exclusion of 751,617 women aged <15 or >24 and 22,078 with missing data, this dataset contained 398,661 young women aged 15–24.

eReferences

1. Corsi DJ, Neuman M, Finlay JE, et al. Demographic and health surveys: a profile. *Int J Epidemiol.* 2012;41:1602–1613.
2. MEASURE DHS/ICF International. Standard recode manual for DHS 6 (version 1.0); description of the Demographic and Health Surveys individual recode data file. Measure DHS, Calverton, Maryland. Calverton, Maryland: MEASURE DHS/ICF International, 2013. Accessed May 21, 2017.
3. Croft, TN, Marshall AMJ, Allen CK, et al. Guide to DHS Statistics. Rockville, Maryland, USA: ICF, 2018. https://www.dhsprogram.com/pubs/pdf/DHSG1/Guide_to_DHS_Statistics_DHS-7.pdf. Accessed Jan 23, 2020.

© 2021 Yang F et al. *JAMA Network Open.*

eFigure 1. Flow Diagram of Selecting the DHS With HIV/AIDS Knowledge Variables Between 2000 and 2018

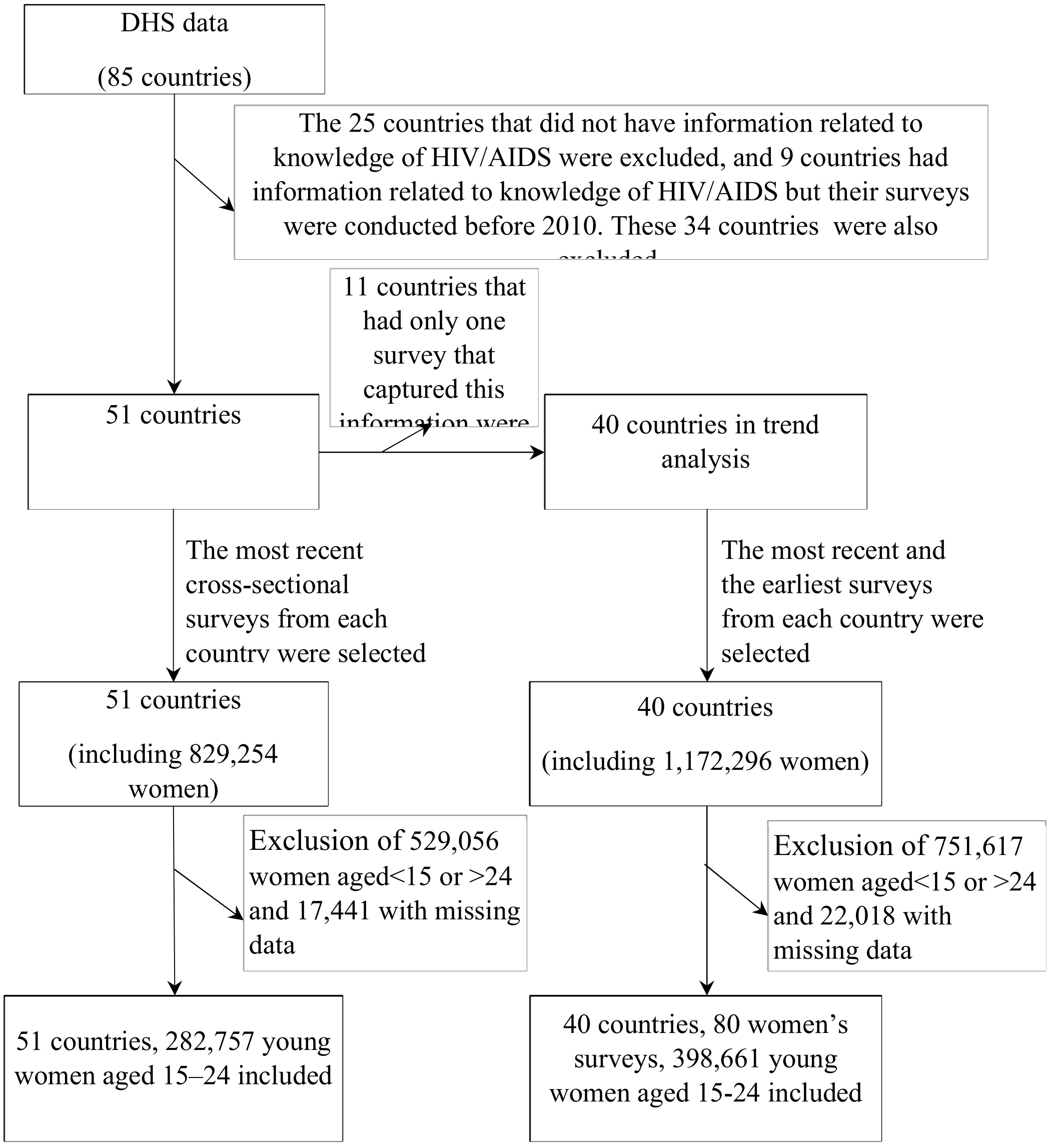

© 2021 Yang F et al. *JAMA Network Open.*

eFigure 2. Disparities in Knowledge of HIV/AIDS by Residential Area Using the Most Recent Surveys

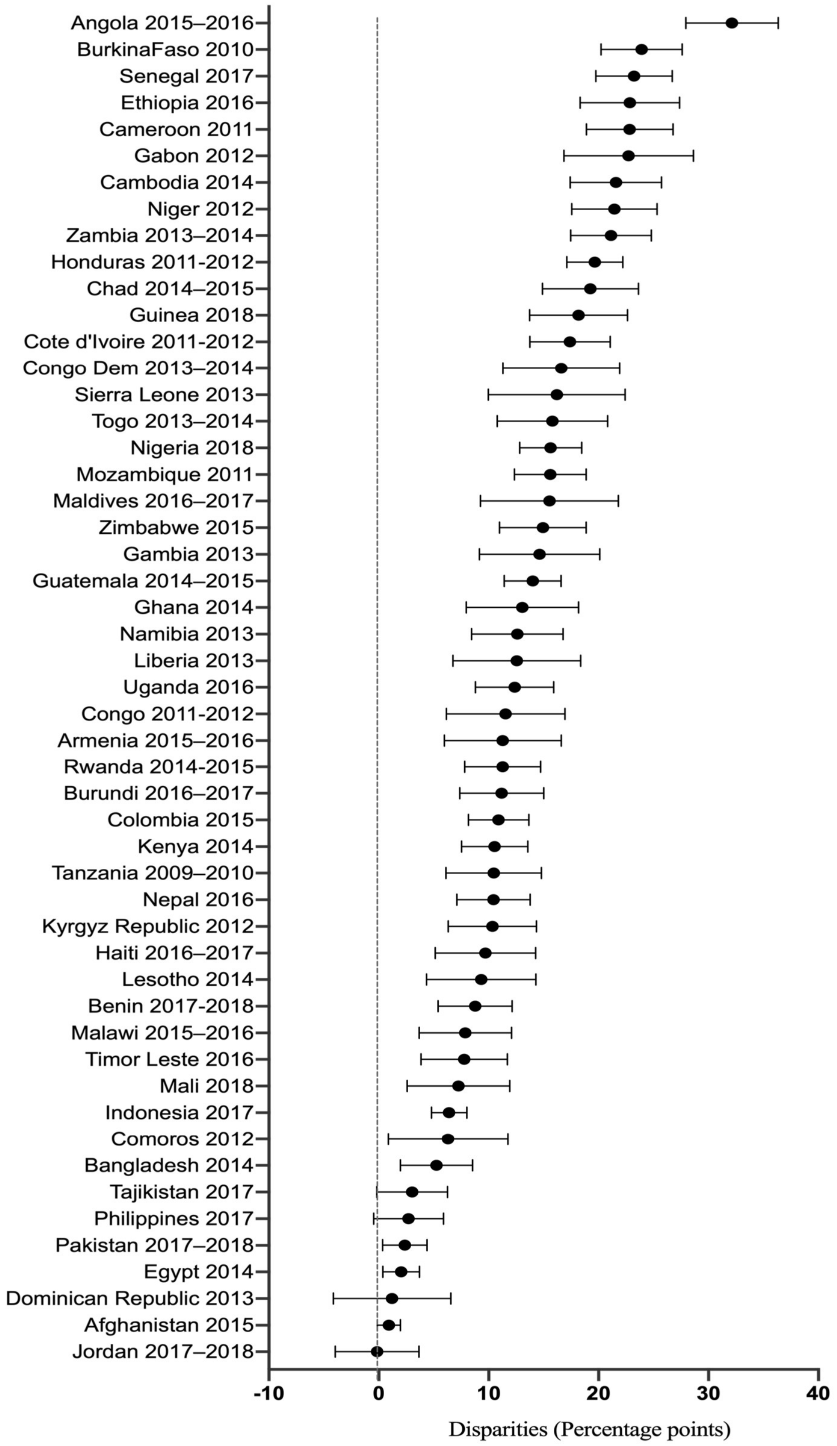

Note: Error bars indicate 95% CIs.

© 2021 Yang F et al. *JAMA Network Open.*

eFigure 3. Disparities in Knowledge of HIV/AIDS by Wealth Quintile Using the Most Recent Surveys

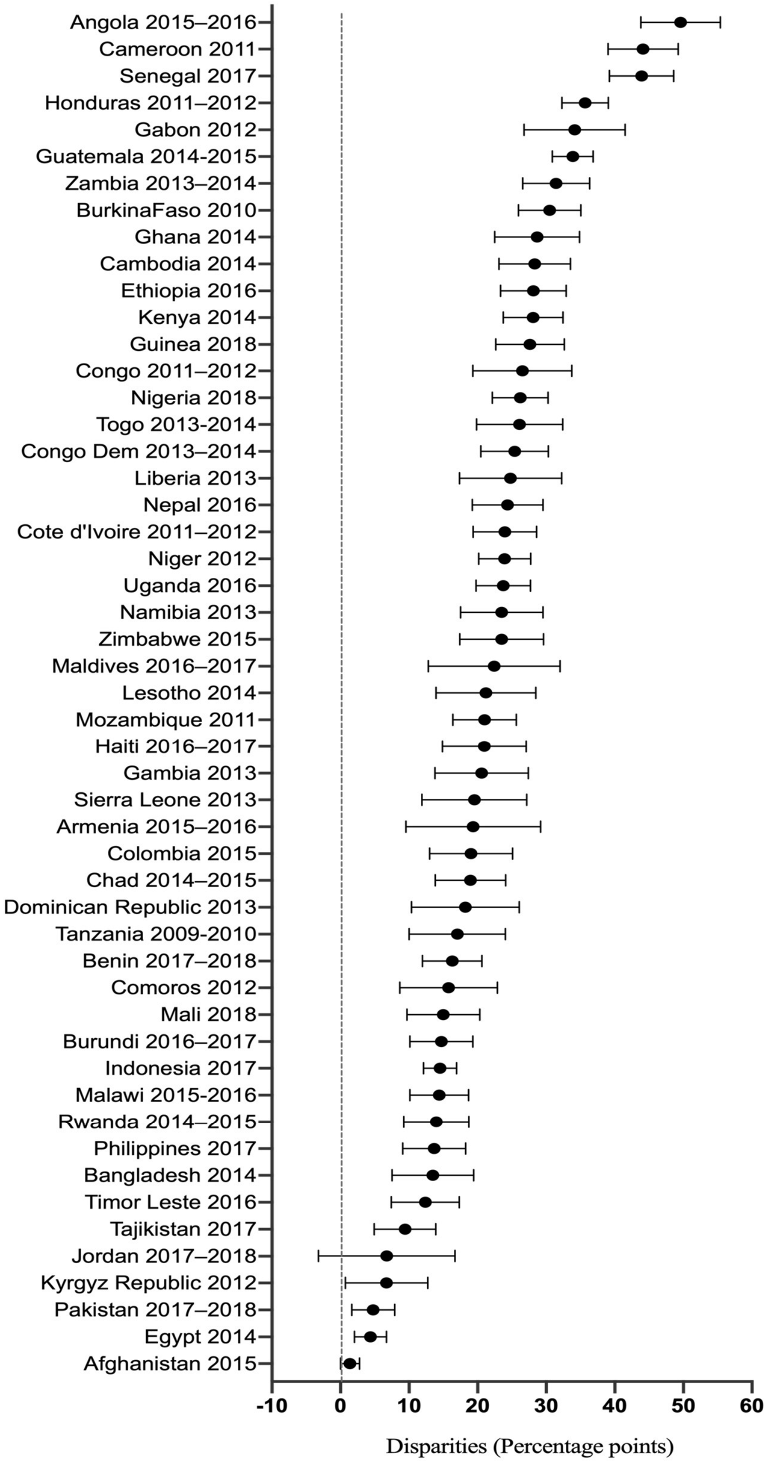

Note: Error bars indicate 95% CIs.

© 2021 Yang F et al. *JAMA Network Open.*

eFigure 4. Disparities in Knowledge of HIV/AIDS by Education Using the Most Recent Surveys

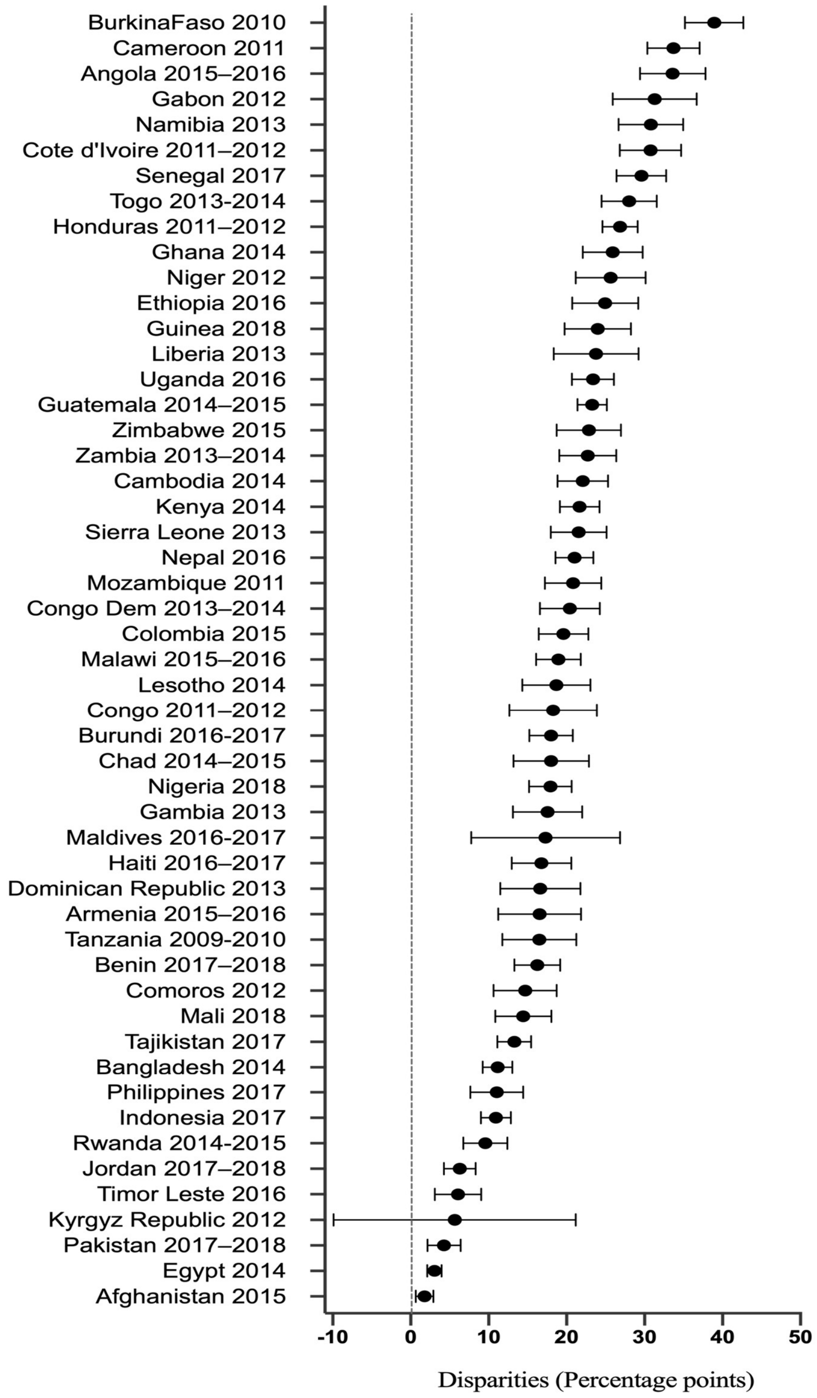

Note: Error bars indicate 95% CIs.

© 2021 Yang F et al. *JAMA Network Open.*

eTable 1. Sample Size and Missing Values in 51 LMICs

Country	Analysis sample size	Missing values		Total missing values
		Knowledge variables	Education variables [a]	
Afghanistan	7,879	33		33
Angola	6,423			
Armenia	1,662	3		3
Bangladesh	5,143	41		41
Benin	3,045	3,206		3,206
Burkina Faso	6,547	40	5	45
Burundi	7,218			
Cambodia	6,042	2		
Cameroon	3,212	3,496		3,496
Chad	2,381	4,503		4,503
Colombia	12,400			
Comoros	2,241	35	6	41
Congo	3,946	17		17
Congo Dem	7,590	71		71
Cote d'Ivoire	3,917	67		67
Dominican	3,541	4		
Egypt	3,783	6		6
Ethiopia	6,401			
Gabon	3,362	45		45
Gambia	4,534	30		30
Ghana	3,325	2		2
Guatemala	10,593	4		4
Guinea	4,267			
Haiti	4,077	2,003		2,003

[a] No missing values for variables for wealth and residential areas

© 2021 Yang F et al. *JAMA Network Open.*

eTable 1. Sample Size and Missing Values in 51 LMICs **(continued)**

Country	Analysis sample size	Missing values		Total missing values
		Knowledge variables	Education variables [a]	
Honduras	9,330	17		17
Indonesia	14,691	75		75
Jordan	2,067			
Kenya	11,437	46		46
Kyrgyz Republic	3,064	41		41
Lesotho	2,842			
Liberia	3,485	14		14
Malawi	10,367			
Maldives	2,133			
Mali	4,116			
Mozambique	5,533			
Namibia	3,560	17		17
Nepal	4,928			
Niger	3,836	23	10	33
Nigeria	15,267			
Pakistan	2,947	1		1
Philippines	9,034			
Rwanda	5,229	23		23
Senegal	7,014		1	1
Sierra Leone	6,693	46		46
Tajikistan	3,850			
Tanzania	4,052	29		29
Timor-Leste	1,807	3,366		3,366
Togo	3,315	22		22

© 2021 Yang F et al. *JAMA Network Open.*

eTable 1. Sample Size and Missing Values in 51 LMICs **(continued)**

Country	Analysis sample size	Missing values		Total missing values
		Knowledge variables	Education variables [a]	
Uganda	8,058			
Zambia	6,635	86	5	91
Zimbabwe	3,938			
Total	282,757	17414	27	17441

© 2021 Yang F et al. *JAMA Network Open.*

eTable 2. Knowledge Level of HIV/AIDS and Associated Socioeconomic Disparities Among Young Women in 51 LIMCs in the Most Recent Survey[a]

Country	Survey years	National level	Residential areas			Wealth group			Education group		
			Urban	Rural	Gap [b]	Richest	Poorest	Gap [b]	Higher education	Primary education or lower	Gap [b]
All countries [c]		29.3(24.1, 34.5)	36.3(30.8, 41.8)	23.4(19.0, 27.9)	12.8(10.6, 14.9)	40.5(34.5, 46.5)	18.6(15.6, 21.5)	21.8(18.3, 25.3)	37.8(31.9, 43.6)	18.4(15.1, 21.8)	19.4(16.6, 22.2)
Afghanistan	2015	1.0(0.7, 1.3)	1.8(0.7, 2.8)	0.8(0.6, 1.1)	0.9(-0.1, 2.0)	2.2(1.0, 3.4)	0.8(0.2, 1.5)	1.4(0.0, 2.8)	2.5(1.5, 3.6)	0.7(0.4, 1.1)	1.8(0.7, 2.9)
Angola	2015–2016	32.5(29.6, 35.5)	41.5(37.9, 45.2)	9.4(7.3, 11.5)	32.1(27.9, 36.3)	57.5(52.1, 62.9)	7.9(5.9, 10.0)	49.6(43.8, 55.4)	48.2(44.2, 52.2)	14.6(12.4, 16.8)	33.6(29.4, 37.8)
Armenia	2015–2016	23.8(20.9, 26.7)	28.7(24.1, 33.3)	17.5(14.8, 20.2)	11.3(6.0, 16.6)	34.4(25.5, 43.2)	15.0(10.8, 19.2)	19.4(9.5, 29.2)	25.4(22.4, 28.5)	8.9(4.0, 13.8)	16.5(11.2, 21.8)
Bangladesh	2014	12.8(11.2, 14.4)	16.7(14.0, 19.3)	11.4(9.4, 13.4)	5.3(2.0, 8.5)	21.2(17.7, 24.6)	7.7(2.9, 12.5)	13.5(7.5, 19.4)	16.7(14.8, 18.6)	5.6(3.9, 7.2)	11.1(9.2, 13.1)
Benin	2017–2018	15.0(13.4, 16.7)	20.1(17.5, 22.7)	11.3(9.2, 13.4)	8.8(5.4, 12.1)	23.6(20.0, 27.2)	7.3(4.8, 9.8)	16.3(11.9, 20.6)	24.7(21.9, 27.5)	8.5(7.0, 10.0)	16.2(13.3, 19.2)
Burkina Faso	2010	31.3(29.5, 33.0)	47.5(44.3, 50.7)	23.6(21.8, 25.4)	23.9(20.2, 27.6)	47.0(43.5, 50.4)	16.5(13.5, 19.4)	30.5(25.9, 35.0)	62.6(59.1, 66.1)	23.6(22.0, 25.3)	38.9(35.2, 42.7)
Burundi	2016–2017	52.5(51.0, 54.1)	62.1(58.7, 65.5)	50.9(49.2, 52.7)	11.2(7.4, 15.0)	59.5(56.8, 62.2)	44.8(41.0, 48.6)	14.7(10.1, 19.3)	62.9(60.9, 65.0)	44.9(42.9, 46.9)	18.0(15.2, 20.8)
Cambodia	2014	37.7(35.7, 39.6)	55.0(51.4, 58.6)	33.4(31.3, 35.5)	21.6(17.4, 25.7)	54.9(51.2, 58.7)	26.6(23.1, 30.0)	28.3(23.1, 33.6)	45.9(43.5, 48.3)	23.8(21.4, 26.2)	22.1(18.9, 25.3)

Abbreviations: LMICs, low- and middle-income countries.

[a] Estimates were weighted and standard errors adjusted for the complex survey design of the demographic and health surveys;

[b] Gap indicates the absolute difference, in percentage points (95%CI), between the two sub-groups: (1) urban area vs. rural area, (2) the richest vs. poorest quintiles, and (3) higher education vs. primary education or lower.

[c] Pooled estimates (Aggregate-level estimates) from random effects meta-analysis, for which the inverse-variance DerSimonian and Laird method was used.

© 2021 Yang F et al. *JAMA Network Open.*

eTable 2. Knowledge Level of HIV/AIDS and Associated Socioeconomic Disparities Among Young Women in 51 LIMCs in the Most Recent Survey **(continued)** [a]

Country	Survey years	National level	Residential areas			Wealth group			Education group		
			Urban	Rural	Gap [b]	Richest	Poorest	Gap [b]	Higher education	Primary education or lower	Gap [b]
Cameroon	2011	34.4(32.4, 36.5)	44.2(41.4, 47.0)	21.4(18.6, 24.1)	22.8(18.9, 26.8)	52.1(48.3, 55.9)	8.0(4.7, 11.3)	44.1(39.0, 49.2)	48.1(45.5, 50.7)	14.4(12.1, 16.6)	33.7(30.4, 37.1)
Chad	2014–2015	11.5(9.8, 13.2)	25.5(21.3, 29.7)	6.2(4.8, 7.7)	19.3(14.9, 23.6)	25.4(21.2, 29.7)	6.5(4.0, 8.9)	19.0(13.8, 24.1)	25.1(21.0, 29.3)	7.1(5.6, 8.6)	18.0(13.2, 22.9)
Colombia	2015	31.9(30.4, 33.4)	34.4(32.5, 36.3)	23.5(21.5, 25.5)	10.9(8.2, 13.6)	41.2(35.5, 46.8)	22.1(20.1, 24.2)	19.0(13.0, 25.1)	33.2(31.6, 34.8)	13.6(10.8, 16.4)	19.6(16.4, 22.8)
Comoros	2012	19.5(17.0, 22.0)	23.8(19.2, 28.4)	17.5(14.5, 20.4)	6.3(0.9, 11.7)	28.9(23.3, 34.5)	13.1(8.9, 17.4)	15.8(8.7, 22.9)	24.5(21.2, 27.8)	9.8(7.1, 12.5)	14.7(10.6, 18.7)
Congo	2011–2012	36.4(33.3, 39.4)	39.6(35.6, 43.6)	28.1(24.4, 31.7)	11.5(6.1, 16.9)	49.8(43.4, 56.3)	23.3(20.0, 26.6)	26.5(19.3, 33.7)	41.1(37.4, 44.9)	22.9(18.6, 27.1)	18.3(12.7, 23.9)
Congo Dem	2013–2014	24.5(21.7, 27.3)	34.3(30.9, 37.6)	17.7(13.5, 21.8)	16.6(11.3, 21.9)	38.4(34.2, 42.5)	13.0(10.3, 15.7)	25.4(20.5, 30.3)	32.9(29.1, 36.7)	12.5(10.5, 14.5)	20.4(16.6, 24.2)
Cote d'Ivoire	2011–2012	20.2(18.1, 22.3)	27.6(24.6, 30.6)	10.2(8.0, 12.4)	17.4(13.8, 21.1)	32.7(29.1, 36.2)	8.7(5.8, 11.6)	24.0(19.4, 28.6)	41.7(38.0, 45.4)	11.0(9.2, 12.7)	30.8(26.8, 34.7)
Dominican Republic	2013	44.7(42.5, 46.9)	45.0(42.5, 47.5)	43.8(39.0, 48.6)	1.2(-4.1, 6.6)	51.7(45.0, 58.5)	33.5(29.1, 38.0)	18.2(10.4, 26.1)	48.4(46.0, 50.8)	31.7(27.1, 36.4)	16.6(11.5, 21.8)
Egypt	2014	2.9(2.2, 3.5)	4.4(2.9, 6.0)	2.4(1.7, 3.1)	2.0(0.4, 3.7)	5.0(2.8, 7.3)	0.7(0.1, 1.2)	4.4(2.0, 6.7)	3.4(2.7, 4.2)	0.4(-0.1, 0.9)	3.1(2.1, 4.0)
Ethiopia	2016	24.3(22.3, 26.3)	41.7(37.6, 45.7)	18.8(16.8, 20.9)	22.8(18.3, 27.4)	39.7(35.9, 43.4)	11.5(8.6, 14.5)	28.1(23.3, 32.9)	42.8(39.0, 46.6)	17.9(15.9, 19.8)	25.0(20.7, 29.2)
Gabon	2012	48.9(45.8, 52.0)	51.3(47.9, 54.8)	28.6(23.8, 33.4)	22.7(16.8, 28.6)	61.9(55.7, 68.0)	27.7(24.2, 31.3)	34.2(26.8, 41.5)	54.0(50.6, 57.4)	22.7(18.4, 27.0)	31.3(25.9, 36.7)
Gambia	2013	26.0(23.2, 28.9)	32.3(27.9, 36.7)	17.7(14.4, 21.0)	14.6(9.1, 20.1)	39.4(33.9, 44.8)	18.8(14.5, 23.1)	20.6(13.8, 27.4)	33.8(29.9, 37.7)	16.3(13.2, 19.3)	17.6(13.1, 22.0)

© 2021 Yang F et al. *JAMA Network Open.*

eTable 2. Knowledge Level of HIV/AIDS and Associated Socioeconomic Disparities Among Young Women in 51 LIMCs in the Most Recent Survey **(continued)** [a]

Country	Survey years	National level	Residential areas			Wealth group			Education group		
			Urban	Rural	Gap [b]	Richest	Poorest	Gap [b]	Higher education	Primary education or lower	Gap [b]
Ghana	2014	32.2(29.6, 34.7)	38.5(35.2, 41.9)	25.5(21.6, 29.3)	13.1(8.0, 18.2)	43.0(37.7, 48.2)	14.3(11.1, 17.4)	28.7(22.5, 34.9)	39.0(36.1, 41.9)	13.1(10.1, 16.1)	25.9(22.1, 29.8)
Guatemala	2014–2015	22.2(20.9, 23.4)	30.2(28.1, 32.3)	16.2(14.7, 17.7)	14.0(11.4, 16.6)	40.6(37.9, 43.2)	6.7(5.4, 8.0)	33.9(30.9, 36.9)	33.2(31.5, 34.9)	9.9(8.8, 11.0)	23.3(21.4, 25.2)
Guinea	2018	20.7(18.5, 22.9)	30.9(27.1, 34.7)	12.7(10.4, 15.1)	18.2(13.7, 22.6)	35.3(30.7, 39.8)	7.6(5.5, 9.7)	27.6(22.7, 32.6)	37.4(33.3, 41.4)	13.4(11.3, 15.4)	24.0(19.7, 28.3)
Haiti	2016–2017	42.0(39.7, 44.2)	47.2(43.8, 50.6)	37.5(34.4, 40.5)	9.7(5.1, 14.3)	51.0(46.4, 55.5)	30.0(25.9, 34.0)	21.0(14.9, 27.1)	47.2(44.5, 50.0)	30.5(27.4, 33.5)	16.8(13.0, 20.6)
Honduras	2011–2012	33.2(31.9, 34.5)	42.2(40.3, 44.2)	22.6(20.9, 24.2)	19.7(17.1, 22.2)	47.7(44.7, 50.7)	12.0(10.4, 13.6)	35.7(32.3, 39.1)	43.7(42.0, 45.5)	16.9(15.4, 18.3)	26.9(24.6, 29.1)
Indonesia	2017	14.6(13.8, 15.4)	17.6(16.4, 18.7)	11.2(10.1, 12.3)	6.4(4.8, 8.0)	21.7(19.9, 23.6)	7.2(5.7, 8.7)	14.5(12.1, 16.9)	15.6(14.7, 16.4)	4.6(2.9, 6.3)	10.9(9.0, 12.9)
Jordan	2017–2018	6.6(5.0, 8.3)	6.6(4.8, 8.4)	6.8(3.4, 10.2)	-0.2(-4.0, 3.6)	12.5(2.5, 22.5)	5.8(3.1, 8.4)	6.7(-3.2, 16.7)	7.2(5.4, 9.0)	0.9(-0.0, 1.9)	6.3(4.3, 8.3)
Kenya	2014	54.5(53.0, 55.9)	60.8(58.4, 63.2)	50.3(48.4, 52.1)	10.5(7.5, 13.6)	63.1(59.9, 66.2)	35.0(32.0, 37.9)	28.1(23.7, 32.5)	65.2(63.2, 67.2)	43.5(41.7, 45.3)	21.7(19.1, 24.2)
Kyrgyz Republic	2012	22.3(20.3, 24.4)	28.7(25.4, 31.9)	18.3(16.0, 20.7)	10.3(6.3, 14.4)	31.9(28.0, 35.9)	25.2(20.7, 29.7)	6.7(0.7, 12.7)	22.4(20.3, 24.4)	16.7(-1.2, 34.6)	5.7(-9.9, 21.2)
Lesotho	2014	37.6(35.3, 39.9)	43.8(39.7, 48.0)	34.5(31.7, 37.3)	9.3(4.3, 14.3)	47.6(42.5, 52.6)	26.4(21.4, 31.3)	21.2(13.9, 28.5)	42.8(40.1, 45.5)	24.1(20.4, 27.8)	18.7(14.3, 23.1)
Liberia	2013	35.8(32.3, 39.2)	40.0(35.0, 45.1)	27.4(24.5, 30.4)	12.6(6.8, 18.4)	46.4(40.1, 52.8)	21.6(17.5, 25.8)	24.8(17.3, 32.2)	49.4(43.8, 55.1)	25.6(22.6, 28.6)	23.8(18.4, 29.2)
Malawi	2015–2016	42.2(40.8, 43.6)	48.7(44.7, 52.6)	40.8(39.3, 42.3)	7.9(3.7, 12.1)	49.9(46.7, 53.1)	35.5(32.7, 38.2)	14.4(10.1, 18.7)	55.3(52.8, 57.8)	36.4(34.8, 37.9)	19.0(16.1, 21.8)

© 2021 Yang F et al. *JAMA Network Open.*

eTable 2. Knowledge Level of HIV/AIDS and Associated Socioeconomic Disparities Among Young Women in 51 LIMCs in the Most Recent Survey **(continued)** [a]

Country	Survey years	National level	Residential areas			Wealth group			Education group		
			Urban	Rural	Gap [b]	Richest	Poorest	Gap [b]	Higher education	Primary education or lower	Gap [b]
Maldives	2016–2017	29.3(26.1, 32.4)	37.0(31.1, 42.9)	21.5(18.9, 24.0)	15.5(9.3, 21.8)	40.9(31.8, 49.9)	18.4(14.6, 22.3)	22.4(12.8, 32.0)	29.5(26.3, 32.7)	12.2(3.1, 21.3)	17.3(7.8, 26.8)
Mali	2018	15.6(13.8, 17.5)	20.8(16.5, 25.0)	13.5(11.5, 15.5)	7.3(2.6, 11.9)	24.0(19.7, 28.3)	9.0(6.0, 11.9)	15.0(9.7, 20.3)	25.2(21.6, 28.7)	10.7(9.0, 12.4)	14.4(10.9, 18.0)
Mozambique	2011	29.8(28.1, 31.5)	39.5(37.1, 41.9)	23.9(21.7, 26.1)	15.6(12.3, 18.9)	41.9(39.3, 44.4)	20.9(17.0, 24.7)	21.0(16.4, 25.6)	44.7(41.7, 47.7)	23.9(21.9, 25.8)	20.8(17.2, 24.5)
Namibia	2013	61.9(59.8, 64.0)	67.7(64.7, 70.6)	55.1(52.1, 58.0)	12.6(8.4, 16.8)	71.5(67.4, 75.6)	48.0(43.6, 52.4)	23.5(17.5, 29.5)	67.8(65.6, 70.0)	37.0(33.1, 40.8)	30.8(26.7, 35.0)
Nepal	2016	20.7(19.1, 22.4)	24.7(22.4, 27.1)	14.3(12.3, 16.3)	10.4(7.1, 13.8)	38.3(34.0, 42.7)	14.0(11.7, 16.3)	24.4(19.2, 29.5)	25.8(24.0, 27.7)	4.8(3.2, 6.5)	21.0(18.6, 23.4)
Niger	2012	13.1(11.6, 14.6)	30.0(26.4, 33.6)	8.6(7.0, 10.1)	21.4(17.5, 25.3)	28.9(25.8, 32.1)	5.0(2.8, 7.2)	23.9(20.1, 27.7)	35.0(30.8, 39.2)	9.3(7.9, 10.7)	25.7(21.2, 30.1)
Nigeria	2018	42.6(41.3, 43.9)	51.4(49.1, 53.6)	35.7(34.0, 37.4)	15.6(12.8, 18.5)	56.3(53.8, 58.8)	30.1(27.2, 33.0)	26.2(22.2, 30.3)	49.6(47.9, 51.3)	31.7(29.7, 33.7)	17.9(15.2, 20.6)
Pakistan	2017–2018	1.8(1.0, 2.6)	3.5(1.6, 5.3)	1.1(0.3, 1.9)	2.4(0.4, 4.4)	5.1(2.0, 8.2)	0.3(-0.3, 0.9)	4.8(1.6, 7.9)	4.6(2.5, 6.7)	0.3(-0.0, 0.6)	4.3(2.2, 6.4)
Philippines	2017	20.2(18.6, 21.7)	21.5(19.2, 23.9)	18.8(17.0, 20.7)	2.7(-0.5, 5.9)	25.3(21.2, 29.3)	11.6(9.7, 13.5)	13.7(9.1, 18.3)	21.0(19.4, 22.6)	9.9(7.3, 12.6)	11.0(7.7, 14.4)
Rwanda	2014–2015	64.9(63.3, 66.5)	73.8(70.8, 76.7)	62.5(60.6, 64.4)	11.3(7.8, 14.7)	71.5(68.5, 74.5)	57.5(53.9, 61.1)	14.0(9.2, 18.7)	70.7(68.5, 73.0)	61.1(59.2, 63.1)	9.6(6.8, 12.4)
Senegal	2017	26.2(24.3, 28.0)	38.1(35.2, 41.0)	14.9(13.0, 16.8)	23.2(19.8, 26.7)	49.6(45.1, 54.1)	5.7(4.4, 6.9)	43.9(39.2, 48.6)	41.4(38.6, 44.2)	11.8(10.1, 13.4)	29.6(26.4, 32.8)
Sierra Leone	2013	29.0(26.0, 32.0)	38.5(33.1, 43.8)	22.3(19.1, 25.5)	16.2(10.0, 22.4)	40.9(34.5, 47.4)	21.4(17.5, 25.4)	19.5(11.9, 27.2)	39.1(35.4, 42.8)	17.5(14.5, 20.5)	21.6(18.0, 25.1)

© 2021 Yang F et al. *JAMA Network Open.*

eTable 2. Knowledge Level of HIV/AIDS and Associated Socioeconomic Disparities Among Young Women in 51 LIMCs in the Most Recent Survey **(continued)** [a]

Country	Survey years	National level	Residential areas			Wealth group			Education group		
			Urban	Rural	Gap [b]	Richest	Poorest	Gap [b]	Higher education	Primary education or lower	Gap [b]
Tajikistan	2017	13.8(11.9, 15.6)	16.1(13.9, 18.3)	13.1(10.7, 15.4)	3.0(-0.2, 6.3)	19.4(16.3, 22.5)	10.0(6.4, 13.6)	9.4(4.9, 13.9)	14.3(12.4, 16.2)	1.0(-0.1, 2.2)	13.3(11.1, 15.5)
Tanzania	2010	49.8(47.7, 51.9)	57.0(53.4, 60.6)	46.5(44.0, 49.1)	10.5(6.1, 14.8)	57.1(53.5, 60.8)	40.1(34.6, 45.6)	17.0(10.0, 24.1)	61.8(57.6, 66.0)	45.3(43.1, 47.5)	16.5(11.8, 21.2)
Timor-Leste	2016	7.7(6.0, 9.3)	12.6(8.9, 16.3)	4.8(3.4, 6.2)	7.8(3.8, 11.7)	14.8(10.2, 19.4)	2.5(0.7, 4.3)	12.4(7.4, 17.3)	8.8(6.8, 10.9)	2.8(0.7, 4.8)	6.1(3.1, 9.1)
Togo	2013–2014	35.1(32.5, 37.6)	43.0(39.0, 47.1)	27.2(24.2, 30.3)	15.8(10.8, 20.8)	45.3(40.3, 50.3)	19.2(15.6, 22.8)	26.1(19.8, 32.4)	47.9(44.7, 51.1)	19.9(17.4, 22.3)	28.0(24.5, 31.6)
Uganda	2016	45.7(44.1, 47.3)	54.7(51.7, 57.8)	42.4(40.6, 44.2)	12.4(8.8, 15.9)	57.6(54.6, 60.7)	33.9(31.4, 36.4)	23.7(19.8, 27.7)	59.9(57.6, 62.2)	36.5(34.9, 38.1)	23.4(20.7, 26.1)
Zambia	2013–2014	44.0(42.1, 45.9)	54.8(51.9, 57.7)	33.7(31.4, 35.9)	21.1(17.5, 24.8)	60.8(57.2, 64.4)	29.3(26.1, 32.5)	31.5(26.6, 36.3)	53.1(50.6, 55.7)	30.4(28.0, 32.8)	22.7(19.1, 26.4)
Zimbabwe	2015	46.3(44.4, 48.3)	55.7(52.7, 58.7)	40.8(38.2, 43.3)	14.9(11.0, 18.9)	57.9(54.1, 61.7)	34.4(29.6, 39.1)	23.5(17.4, 29.6)	51.4(49.3, 53.5)	28.5(24.8, 32.2)	22.9(18.7, 27.0)

© 2021 Yang F et al. *JAMA Network Open.*
